# Exogenous application of the plant signalers methyl jasmonate and salicylic acid induces changes in volatile emissions from citrus foliage and influences the aggregation behavior of Asian citrus psyllid (*Diaphorina citri*), vector of Huanglongbing

**DOI:** 10.1371/journal.pone.0193724

**Published:** 2018-03-29

**Authors:** Joseph M. Patt, Paul S. Robbins, Randy Niedz, Greg McCollum, Rocco Alessandro

**Affiliations:** United States Department of Agriculture, Agricultural Research Service, U.S. Horticultural Research Laboratory, Fort Pierce, Florida, United States of America; Fujian Agriculture and Forestry University, CHINA

## Abstract

Huanglongbing, also known as citrus greening, is a destructive disease that threatens citrus production worldwide. It is putatively caused by the phloem-limited bacterium *Candidatus* Liberibacter asiaticus (Las). Currently, the disease is untreatable and efforts focus on intensive insecticide use to control the vector, Asian citrus psyllid (*Diaphorina citri*). Emerging psyllid resistance to multiple insecticides has generated investigations into the use of exogenously applied signaling compounds to enhance citrus resistance to *D*. *citri* and Las. In the present study, we examined whether foliar applications of methyl jasmonate (MJ), a volatile signaling compound associated with the induced systemic resistance pathway, and salicylic acid, a constituent of the systemic acquired resistance pathway, would elicit the emission of defense-related volatiles in citrus foliage, and what effect this might have on the host-plant searching behavior of *D*. *citri*. Comparisons were made of volatiles emitted from growing shoots of uninfected and Las-infected ‘Valencia’ sweet orange (*Citrus sinensis*) trees over two consecutive sampling days. A settling behavioral assay was used to compare psyllid attraction to MJ-treated vs. Tween-treated citrus sprigs. All three main effects, Las infection status, plant signaler application, and sampling day, influenced the proportions of individual volatile compounds emitted in different treatment groups. MJ- and SA-treated trees had higher emission rates than Tween-treated trees. Methyl salicylate (MeSA) and β-caryophyllene were present in higher proportions in the volatiles collected from Las-infected + trees. On the other hand, Las-infected + MJ-treated trees emitted lower proportions of MeSA than did Las-infected + Tween-treated trees. Because MeSA is a key *D*. *citri* attractant, this result suggests that MJ application could suppress MeSA emission from Las-infected trees, an approach that could be used to discourage psyllid colonization during shoot growth. MJ application enhanced emission of E-β-ocimene, indole, volatiles attractive to many of the psyllid’s natural enemies, indicating that MJ application could be used in an ‘attract and reward’ conservation biological control strategy. Volatile emissions in SA-treated trees were dominated by MeSA. MJ application elicited aggregation behavior in *D*. *citri*. Similar numbers of psyllids settled on MJ-treated versus Tween-treated sprigs, but a significantly greater percentage of the MJ-treated sprigs had aggregations of nine or more psyllids on them. Taken together, the results of this study indicate that exogenous applications of MJ or SA could be used to influence Asian citrus psyllid settling behavior and attract its natural enemies.

## Introduction

Huanglongbing also known as citrus greening disease, is a devastating disease that threatens commercial citrus production worldwide [1]. It is likely caused by *Candidatus* Liberibacter asiaticus’ (Las) [2, 3]. Las inhabits the phloem of plants in the genus *Citrus* and a few closely related genera, such as *Murraya* and *Bergera*. Infected trees may remain asymptomatic for several years but eventually develop severe chlorosis and die [2,3]. Because it has not yet been possible to develop *in vitro* cultures of Las, therapeutic treatments are lacking and major regional citrus industries have experienced significant losses due to the intractability of the disease. For example, in Florida, production has decreased over 40% since 2004 and between 2012 and 2016 there has been a loss of $4.4 billion in cumulative industry output and a loss of 7,945 fulltime and part-time jobs [4,5].

Las is transmitted by the Asian citrus psyllid (*Diaphorina citri* Kuwayama (Hemiptera: Psyllidae)) [6–8]. These tiny insects feed only on the phloem of *Citrus* and closely related genera. As the psyllid feeds, the pathogen is transferred from the salivary glands through the stylet into the phloem cells. *D*. *citri* mates and oviposits only on young growing shoots where its immatures can take advantage of the tenderness of the cuticle and the relatively higher levels of nitrogen in the phloem. As a vector, *D*. *citri* is highly problematic because it reproduces rapidly and can disperse over considerable distances in relatively short periods of time [9]. Growers rely on intensive use of chemical insecticides to suppress psyllid populations, [10–11], but even with persistent and diligent applications, huanglongbing has continued to spread. Emerging regulatory restrictions on certain insecticide classes, such as neonicotinoids, and the appearance of resistance to multiple chemical classes in some psyllid populations [12] emphasizes the need to develop alternative control strategies. The widespread adoption of chemical insecticides against *D*. *citri* precludes effective biocontrol strategies from being accepted, though studies have shown that arthropod predators and parasitoids can suppress *D*. *citri* [13–16].

Citrus leaves have numerous glands [17] that produce essential oils that are rich in mono- and sesquiterpenes [18] and which function as a constitutive defense against microbial attack [19]. In addition to the protection afforded by the oil glands, induced defenses in citrus can be triggered by damage caused by pathogens and herbivores; signaling compounds appear to play an important role in these processes. For example, in clementine oranges (*Citrus* x *clementina*), spider mite feeding elicited production of stress- and protection related proteins within 72 hours following infestation [20]. In the same study, exogenous application of the plant signaler methyl jasmonate (MJ) also induced the production of many of these same proteins. Following inoculation with conidia of the pathogenic fungus *Alternaria alternata*, the leaves of rough lemon (*C*. *jambhiri* Lush) contained elevated levels of defense related genes relative to uninfected controls [21]. The authors found that the gene responsible for the production of epoxide hydrolase, an enzyme responsible for strengthening the cuticle against fungal infection, was induced following exposure to MJ and the C6 volatiles (*E*)-2-hexenol and (*Z*)-3-hexenol.

Infection by Las profoundly disrupts the metabolism of citrus trees [22] and induces pronounced changes in the foliar volatile profile [23–28]. Importantly, *D*. *citri* is more attracted to the odor emitted by Las-infected foliage than to that of healthy foliage [23, 27–28]. Mann et al. [23] found that Las-infected leaves emitted reduced amounts of limonene and methyl anthranilate, the dominant constituents of uninfected foliar odor, but high amounts of methyl salicylate (MeSA). This volatile is emitted following activation of the Systemic Acquired Resistance (SAR) pathway, which is triggered in response to invasion by phytopathogens and the feeding activities of sap-sucking insects [29–30]. Mann et al. [23] found that nutrient levels were reduced in the phloem sap of Las-infected trees, and retention of *D*. *citri* on infected trees waned after several days, a period of time sufficient for the psyllids to acquire Las and then disperse.

Mann et al. [23] suggest that the Las-*D*. *citri*-*Citrus* pathosystem is an example of the deceptive host phenotype hypothesis [31]. That is, by altering the foliar odor of its host plant, Las mediates the behavior of its vector to promote its own dispersal from nutritionally inferior, infected hosts to uninfected hosts. Parallel situations have been found in other psyllid-host plant-pathogen systems. For example, apple trees infected with apple proliferation phytoplasma (*Candidatus* Phytoplasma mali) emit β-caryophyllene, which attracts its vector, *Cacopsylla picta* (Foerster) a psyllid that reproduces on apples [32–35]. The psyllid is more attracted to the odor emitted by the foliage of infected trees than to the odors emitted by healthy trees. Davis et al. [36] likewise found that the foliar odor of potato plants (*Solanum tuberosum* L.) infected with zebra chip (*Candidatus* Liberibacter solanacearum) differed from that of healthy plants, and that potato psyllids (*Bactericera cockerelli* Sulc.) initially settled on plants exposed to infected psyllids but later defected and oviposited primarily on plants exposed only to uninfected psyllids. This was similar to the findings of Mann et al. [23] in that infected plants initially recruited psyllids but later repelled them. Interestingly, *Tamarixia radiata* (Waterson), a nymphal parasitoid of *D*. *citri*, is attracted to MeSA and uses it as a cue to locate plants infested with psyllids [37], adding yet another trophic dimension to the role of MeSA in this system.

Psyllid infestation by itself also changes the composition of foliar volatiles and psyllid response [24,27]. As the psyllid’s stylet probes the cortex seeking a vascular bundle, it secretes a salivary sheath that helps digest the tissue. Although probing causes only minor damage, citrus tissue is responsive to it, possibly via interactions with components of the salivary sheath. After Valencia sweet orange (*C*. *sinensis)* was infested with *D*. *citri*, the relative amount of 21 out 27 volatile compounds extracted from the leaves increased two- to 10-fold relative to that of uninfested foliage [24]. MeSA emitted by psyllid-infested plants may function as a kairomone that alerts psyllids to the presence of conspecifics on those plants [27].

Collectively, these studies highlight the dynamism of Las-*D*. *citri*-*Citrus* pathosystem. There is current interest in using exogenously applied signaling compounds to enhance the citrus resistance to *D*. *citri* and Las. For example, applications of β-aminobutyric acid (BABA), a non-protein amino acid that has a control effect on many plant pathogens and primes both SA-dependent and independent pathways, impacted the survivorship of *D*. *citri* eggs, nymphs, and adults in potted citrus trees [38]. BABA application resulted in elevated levels of proteins antagonistic to pathogens, reduced levels of certain macro- and micronutrients were also reduced relative to control trees. The decreased nutrient levels in Las-infected foliage were thought to instigate the departure of *D*. *citri* from infected trees, as was observed in Las-infected trees by Mann et al. [23]. Martini et al. [28] showed that exposing *D*. *citri* to MeSA could inhibit their ability to colonize Las-infected trees.

Damage to plant tissue caused by psyllids and other homopteran insects has typically been thought to elicit the systemic acquired resistance pathway [39–42]; most of the studies of signaler compounds and inducible response in citrus to *D*. *citri* have revolved around this defense pathway. Induced systemic resistance (IRS), the other principle inducible plant defense pathway, understandably has not received as much attention as a potential interacting agent with this pathosystem. In the present study, we examined whether foliar applications of methyl jasmonate (MJ), a volatile signaling compound that induces the induced systemic resistance pathway [43–45], would elicit the emission of defense-related foliar volatiles in citrus, and what effect this might have on the host plant searching behavior of *D*. *citri*. Because Las has been observed to influence volatile emission from infected plants, both Las-infected and non-infected trees were tested with MJ. For the sake of comparison, we also examined the volatile profile of trees sprayed with salicylic acid (SA), one of the primary volatile signaler compounds associated with the SAR pathway.

## Materials and methods

### Plant material

Tests were conducted on ‘Valencia’ sweet orange (*Citrus sinensis* L. Osbeck) scions grafted onto US-942 rootstocks. The grafted trees were 12 to 18 months old. ‘Valencia’ sweet orange was selected because it is one of the main varieties grown in Florida for juice production and is considered to be highly susceptible to Las infection [46,47]. To produce the Las-infected trees, a selection of the ‘Valencia’/US-942 trees were graft inoculated with buds from Las-infected ‘Valencia’ trees, which had been validated as infected by qPCR testing [48]. The graft-inoculated trees were grown for 3 months. Individual leaves were then collected and assayed by qPCR to verify and quantify the systemic Las infection titers in each tree. All Las-infected trees used in the studies had C_t_ values of 22–24. Trees were grown in a greenhouse maintained at ca. 15–30°C under a natural light regime. Trees were watered daily to every other day on an as needed basis and fertilized with a 20-10-20 fertilizer (J. R. Peters Inc., Allentown, Pennsylvania. USA) at 325 ppm once each week.

### Application of MJ and SA

Solutions of methyl jasmonate and salicylic acid (Sigma-Aldrich, St. Louis, Missouri, USA) were prepared by mixing these compounds in a 0.1% (v/v) aqueous solution containing TWEEN^®^ 80 (Sigma-Aldrich, St. Louis, Missouri, USA), an emulsifier used to improve dispersion of the test compounds. The control spray consisted of the 0.1% aqueous TWEEN^®^ 80 solution alone = Tween-treated). Preliminary dose-response tests indicated that a 10 mM solution of MeJA and 7 mM solution of SA were optimal concentrations for eliciting induction of volatiles from ‘Valencia’ sweet orange foliage.

For each test, two uninfected (Las-) and two infected (Las+) trees were selected. The selected trees had been lightly pruned 2 weeks earlier so that numerous flushing shoots were present at the time of testing. A pair of Las- and Las+ trees were sprayed with the control (Tween) solution whereas the other pair of trees were sprayed with either the MeJA or SA solution. Sprays were applied to the point of runoff with a 1L hand sprayer. Prior to beginning the collection of volatiles, the sprayed foliage was allowed to air-dry, a process that typically lasted ca. 1 h. A total of five replicated tests were conducted for each treatment.

### Foliar volatile collection

Volatiles samples were collected using a headspace-trap and purge method. During sampling, the trees were arranged in a row on a greenhouse bench. The control sprayed trees were positioned upwind of the MeJA- and SA-treated trees to reduce potential effects to the control individual of induced volatiles emanating from the treated individuals. Samples were collected simultaneously from single flushing terminal shoots on each of the four treated trees. The headspace vessel consisted of a 2000 mL, 3-neck flask with two ground glass 24/40 side openings and a 45/50 ground glass center opening (Ace Glass Inc., Vineland, New Jersey USA). The flask was adapted by removing the center 45/50 fitting with rotary tool equipped with a diamond cutting bit (Harbor Freight Tools, Camarillo, California, USA). The removal of this fitting enlarged the center opening such that the shoot could be easily introduced into the flask without damaging the leaves. After insertion of the shoot, several cotton balls were gently placed around the stem to loosely seal the center flask opening.

Headspace volatiles were collected using glass-lined stainless steel tubes (3.0 mm ID) (Scientific Instrument Services, Ringoes, New Jersey, USA) containing 100 mg of PoraPak Q (Waters Corporation. Milford, Massachusetts, USA) and plugged with silane-treated glass wool (Supelco, Bellefonte, Pennsylvania, USA). One end of the sorbent tube was inserted into a 24/40 male adapter and secured with a bevel-seal threaded fitting. The other end was screwed into a stainless steel adapter that was connected to a length of corrugated PTFE tubing (Tef-Cap Industries, Westchester, Pennsylvania, USA). This assemblage was then placed into one of the flask’s side openings and locked with a plastic clip. The PTFE tube was attached to a personal air-sampling pump (SKC Corporation, Eighty Four, Pennsylvania, USA) with a flow rate of 100 ml/min. To reduce potential background contaminates present in the greenhouse air, clean air was introduced into the headspace flask from a pressurized air cylinder through a PTFE tube inserted into a male adapter in the flask’s second side opening. A slightly positive pressure was maintained in the headspace flask by introducing the makeup air at a flow rate of 108–110 ml/min. Flow rates were verified with a flow calibrator (Mesa Labs, Butler, New Jersey, USA). A 100 ml three-neck flask through which air from the pressured cylinder was circulated but which did not contain any plant material was also included in each test as a control for background air contamination.

The headspace was sampled continuously for 4 h, from 10:00 until 14:00, on two consecutive days. At the end of the sampling period, the sorbent tubes were removed, capped, and stored at -4°C. Air from the pressurized cylinder circulated continuously in the flasks between sampling periods to prevent contamination from greenhouse air, maintain shoot vigor and prevent the condensation of foliar volatiles and transpiration vapor. An overhead shade cloth prevented direct sunlight from overheating the headspace flasks. To maintain a minimum level of light during the tests, the plants received additional illumination from T-4 fluorescent tubes (54 W 6400K; AgroMax; https://growagromax.com/products/4ft-pure-series/) and compact fluorescent bulbs (125 W, 6500 K; (Felizworld; http://www.felizworld.com). The supplemental lights were turned on at 9:00 and turned off at 17:00 during the sampling period. At the end of the collection period, each sampled sprig was cut at the level of the bottom of the collection flask. The sprigs were dried overnight in an oven at 40°C and then weighed for calculating emission rates.

### Foliar volatile analysis

Sorbent tubes were stored for 12- to 72 h prior to elution. The sorbent tubes were eluted with a 2 ml aliquot of a 1:9 (v/v) acetone: hexane mixture. Following elution, the elutant was evaporated under a gentle stream of nitrogen to a volume of 25 μl, determined by weight. A 1μl aliquot of the elutant contained 50 ng of toluene added as an internal standard.

One microliter of the elutant was injected into an Agilent 6890 Series gas chromatograph equipped with a mass selection detector (Agilent 5973) (Agilent Technologies, Santa Clara, California, USA) under the following conditions: injector: 250°C, split-less mode; head pressure: 16.8 PSA; flow rate: 1.1 ml/min; column: 60 m by 0.25 mm HP-5MS methyl silicon (J & W Scientific, Folsom, California, USA), column thickness 0.25 μm; temperature program: 35°C (4 min) then 9°C/min to 80°C (4 min) then 5°C/min to 150°C (5 min) then 5°C/min to 250°C (6 min). The mass spectra detector parameters were as follows: transfer line temperature: 280°, mass range scanned: 20–400 mass units, source temperature: 280°, MS quad temperature: 280°, and EM volts: 2,188.

Spectral matching was accomplished with Chem-Station software (Agilent Technologies, Santa Clara, California, USA) and the NIST 98 Mass Spectral Library (v 1.7) and Adams Essential Oil Components Library (Allured Publishing, Carol Stream, Illinois, USA). A positive matched required a spectral fit of ≥ 90. Verification of compound identification was obtained by comparison of their spectra to the retention times and mass spectra of commercially available standard compounds (Sigma-Aldrich, St. Louis, Missouri, USA). In cases where standard compounds were not readily available, natural product essential oils (Ananda, Boulder, Colorado, USA) known to contain the compounds in question [18] were used to verify the retention times and mass spectra of sample components. Single standard compound 1.0 μL injections contained 20 ng standard/μL hexane and essential oil 1.0 μl injections contained 50 ng essential oil/μL hexane. The concentration of individual components was determined by comparison of their peak area with that of the internal standard (toluene) added to the elutant. This concentration was then used to calculate the percentage of each component with respect to the total amount of each sample.

### Psyllid aggregation behavior assay

A settling assay [49] was used to compare the aggregation behavior of Asian citrus psyllid on flushing citrus sprigs treated with MJ versus Tween-treated, control sprigs. For this experiment, sprigs were excised from potted *Citrus macrophylla* (Wester) since the experimental psyllids were reared on this plant and *D*. *citri* responds behaviorally more strongly to its natal host plant than to novel host plants [50,51]. Details of the assay procedure are given in Hall et al. [49]. In brief, two freshly-cut *C*. *macrophylla* sprigs, each ca. 2.5 cm long, were placed in individual vials (25 dram clear-plastic vials, 39 mm diameter x 85 mm high (BioQuip Products, Inc., Gardenia, California, USA) capped with white snap-on plastic lids. Each vial cap was equipped with a 6 mm hole to permit psyllid entry into the vial. Once inside the vial, psyllids strongly tended to feed and remain on the sprigs. For each assay, nine vials were placed in an incubator fitted with fine screening to confine the psyllids in an area ca. 20.5 cm high x 67.0 cm wide x 41.5 long. The vials were arranged in two rows directly underneath fluorescent lights positioned 13.5 cm above the vial caps. 200 adult psyllids, 5 to 10 days old (post-eclosion), were released into each incubator at the start of the test. After 24 h, the vials were sealed and placed in a freezer for two hours. The numbers of psyllids in each vial were then counted and their gender recorded. A vial was considered to have an aggregation if ≥ 9 psyllids were present. Approximately 120 min prior to the start of each test, MJ-treated sprigs were sprayed with a 10 mM MJ solution as described in the section on MJ application above. Control sprigs were sprayed with a 0.1% aqueous solution of TWEEN 80. The sprigs were allowed to air dry prior to excision and placement into the vials. The cut ends of the sprigs were placed into 1.3 mL Eppendorf vials filled with tap water; the Eppendorf tubes were held in place with plastic holders glued to the inside of the vial. No-choice tests, meaning that all of the sprigs presented within an individual incubator were either MJ-treated or Tween-treated, were performed to prevent volatilized MJ from treated sprigs from contaminating Tween-treated sprigs. At least one MJ-treated and one control tests were run simultaneously, in some cases two MJ-treated tests were run simultaneously with a single control test. A total of eight replications were performed for the control tests and a total of 11 replications were performed for the MJ-treated test.

### Statistical analysis

A 3-factor 2-level factorial experiment was conducted to determine the effects of signaler compounds (MJ or SA), Las infection status (+, -), and collection day (day 1, day 2) on the proportional composition of volatile compounds and on individual chemical classes. The data, the proportion of each component within a given sample, were square root transformed to reduce the effect of the most abundant volatiles. A resemblance matrix was constructed using the Bray–Curtis similarity measure [52], a procedure that differentiates compositional profiles and takes into account relative component magnitudes. Main and interaction effects were determined by permutational multivariate analysis of variance (PERMANOVA) using 10,000 permutations, and an analysis of similarity (ANOSIM). ANOSIM is multivariate procedure that determines the strength and significance of factor level groups. To visualize differences in the effects of Las infection status, signaler compound, and sampling day on volatile composition, non-metric multi-dimensional scaling (NMDS) plots, based on the generated similarity matrices, were constructed. A ranking of each volatile compound’s contribution between groups was obtained using a similarity percentage breakdown procedure (SIMPER). All analyses were conducted using Primer 7 software [53]. Mean emission rate, expressed as ng of volatile compound collected per mg dry weight of shoot sample per hour of collection, were compared with planned t-tests with *P* ≤ 0.0125. For the settling behavior assay, the mean numbers of psyllids per vial per test were compared by t-test, and the proportion of vials with aggregations (≥ 9 individual psyllids/vial) per test was compared by G-test.

## Results

### Effects of methyl jasmonate application

**Fig 1:**
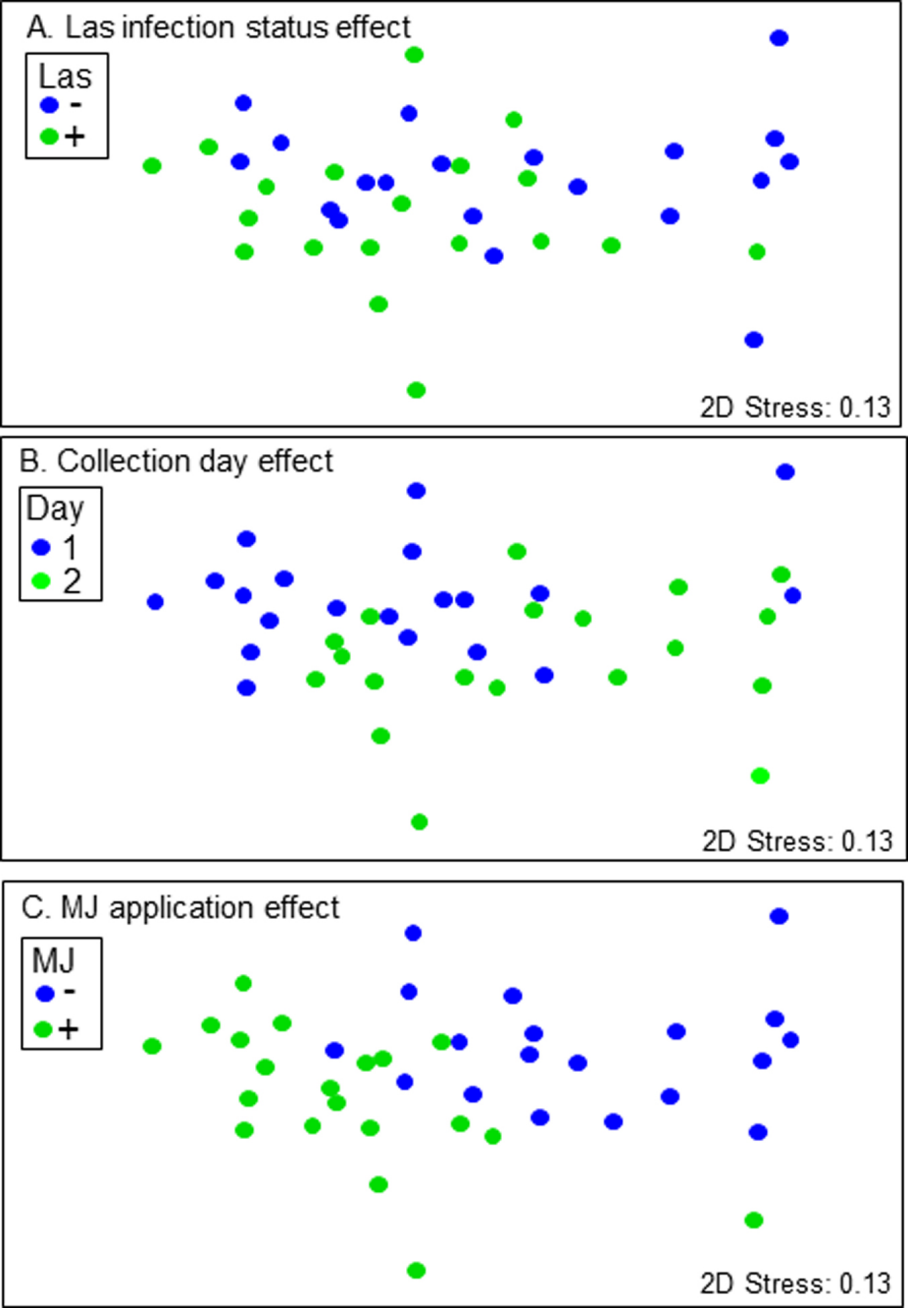
Nonmetric multidimensional scaling (NMDS) of the main effects on proportions of emitted volatile compounds in the MJ experiments. NMDS plots showing the effects of (A) Las infection status, (B) MJ application, and (C) collection day on the proportions of volatiles emitted. Comparisons of the proportions of volatile compounds in each treatment were based on Bray-Curtis similarities plotted in a non-metric multidimensional scaling.

On the first day of sampling, a total of 32 and 33 volatile compounds were collected, respectively, from growing shoots of Tween-treated + uninfected and Tween-treated + Las-infected trees (Table 1). In comparison, a total of 36 and 43 volatile compounds were collected, respectively, from the foliage of uninfected + MJ-treated and Las-infected + MJ-treated trees. On the second day of sampling, 25 to 29 hours post MJ application, the total numbers of volatiles emitted in all treatments decreased relative to the totals emitted on day 1 (Table 1). Although the foliage of uninfected + Tween-treated and Las-infected + Tween-treated trees emitted similar numbers of volatiles compounds, the mean emission rate of the latter (0.44 ng volatiles/mg foliage/h) was twice as great as the former (0.21 ng volatiles/mg foliage/h) (Table 1; S1 Appendix). MJ treatment elicited high volatile emission rates with Las-infected trees having a significantly higher mean emission rate (1.26 ng volatiles/ mg foliage/h) than Tween-treated trees. The emission rates in all treatments decreased on day 2 of sampling. Permutational MANOVA showed that three main effects, namely Las infection status, MJ application, and sampling day, strongly influenced volatile emission (S1 Table); MJ application was the strongest effect. The MJ x sampling day interaction effect was also significant, but not as influential as the main effects alone. The analysis of similarities procedure (ANOSIM) indicated that Las infection and collection day moderately influenced volatile composition (Las infection status: ANOSIM global R = 0.245, *P* ≤ 0.002 (Fig 1A); collection day: ANOSIM global R = 0.336, *P* ≤ 0.0003 (Fig 1B)), while MJ treatment had a very strong effect (ANOSIM global R = 0.614, *P* ≤ 0.0001 (Fig 1C)). Note that the global R test statistic indicates the relative separation among *a priori* defined groups and is based on differences of mean ranks among and within groups. R values closer to 1.000 indicates that samples within groups are more similar to each other than to samples from a different group, whereas R values closer to of 0.000 indicate that samples are grouped more randomly [53]. The stress values shown in the NMDS plots signify how well the two-dimensional plot represents relationships among samples in multidimensional space, with stress values below 0.15 indicating a good fit [53].

**Table 1:** Methyl jasmonate experiment results showing mean absolute amounts, emission rates, and relative proportions of volatile compounds collected on two consecutive days from Valencia sweet orange (infected with Las or uninfected; treated with methyl jasmonate or Tween-treated).

		Day 1				Day 2			
Compounds	RT	Las- MJ-	Las+ MJ-	Las- MJ+	Las+ MJ+	Las- MJ-	Las+ MJ-	Las- MJ+	Las+ MJ+
**No. of compounds**		32	33	36	43	19	27	32	36
**Amount collected (ng)**		292	628	1243	1526	124	232	547	565
Emission rate (ng/mg/h)*		0.21a	0.44ab	1.08bc	1.26c	0.08A	0.15AB	0.44B	0.39AB
*Aliphatic Aldehydes*									
Heptanal	14.58	0.9	-	-	-	0.7	-	-	-
**Octanal**	**18.56**	**3.4**	**2.2**	**1.3**	**0.5**	**4.9**	**2.9**	**2.5**	**1.6**
**Nonanal**	**22.32**	**35.5**	**17**	**10.7**	**5.4**	**33**	**25.6**	**15.3**	**11.8**
**Decanal**	**25.73**	**9.9**	**6.4**	**3.8**	**2.1**	**18.3**	**6.7**	**-**	**5**
Undecanal	28.97	1.8	0.8	0.6	0.4	2.4	1.2	1	0.7
Dodecanal	32.94	0.5	0.4	0.3	0.1	2.2	0.1	0.2	0.2
*Aromatics*									
Benzyl alcohol	19.8	0.2	0.2	0.1	0.1	-	-	-	-
**Methyl Salicylate**	**25.58**	**0.6**	**4**	**0.3**	**0.5**	**0.9**	**8.1**	**6.8**	**1.2**
*Green leaf volatiles*									
Z-3-Hexenyl acetate	18.68	0.1	0.6	0.1	0.2	-	-	0.2	0.4
*Monoterpenes*									
α-Pinene	16.03	1.3	0.8	0.5	0.5	10.3	1.4	1	0.7
Sabinene	17.58	3.6	6.7	3	4.1	2.5	4.2	2.7	2.1
β-Pinene	17.76	0.9	1.5	0.9	0.6	-	1.9	2.2	1.4
**Myrcene**	**18.13**	**1.6**	**2.5**	**2.5**	**1.4**	**-**	**0.6**	**0.9**	**2**
α-Phellandrene	18.77	0.2	-	-	0.2	-	0.3	0.5	-
δ-3-Carene	19	1.2	1.9	1	1.6	1.1	1.1	1.4	0.7
α-Terpinene	19.23	0.1	0.5	0.1	0.3	0.2	0.9	0.3	0.3
p-Cymene	19.52	0.3	1	0.4	0.4	2.9	2.6	0.9	1
**Limonene**	**19.69**	**3.5**	**3.6**	**1.5**	**2.5**	**1.2**	**2.4**	**1.1**	**0.8**
**Z-β-Ocimene**	**19.90**	**0.8**	**1.2**	**1.8**	**2**	**0.3**	**0.5**	**8.3**	**1**
**E-β-Ocimene**	**20.37**	**26.2**	**36.8**	**59.8**	**59.1**	**15**	**21.3**	**41.1**	**29.2**
ϒ-Terpinene	20.81	0.5	0.9	0.4	0.4	0.6	2.1	0.5	0.8
E-Sabinene hydrate	21.12	0.2	0.5	0.4	0.5	-	0.6	0.4	0.3
Terpinolene	21.9	0.5	0.6	0.2	0.5	0.2	0.7	0.3	0.3
Linalool	22.17	0.4	1.6	0.6	0.8	-	-	0.2	-
allo-Ocimene	23.22	0.5	0.8	1.2	1.1	-	0.3	1	0.8
1,3,8-p-Menthatriene	23.31	0.1	-	0.1	0.1	-	-	-	-
Terpinen-4-ol	25.02	0.2	-	-	0.2	-	0.2	-	0.1
α-Terpineol	25.42	-	-	-	0.2	0.2	-	1.2	0.1
Neral	26.91	-	-	-	0.2	-	-	-	-
Car-3-en-2-one	27.42	-	-	-	0.1	-	-	tr	0.1
Geranial	27.81	-	-	0.2	0.5	-	-	-	-
Geranyl acetone	34.73	-	0.6	0.1	0.1	-	0.6	0.2	0.1
*N-compounds*									
Methyl-butyl aldoxime isomers	12.74	-	-	-	0.12	-	-	-	0.17
Benzyl cyanide	28.74	-	-	-	-	-	-	0.2	0.3
**Indole**	**28.74**	**0.1**	**0.4**	**4.5**	**3.4**	**-**	**-**	**0.9**	**2**
*Oxylipins*									
Z-Jasmone	32.82	-	-	0.5	0.5	-	-	tr	tr
*Sesquiterpenes*									
β-Elemene	32.65	-	0.8	0.2	0.4	-	-	-	-
**Caryophyllene**	**33.98**	**2.8**	**4.7**	**2**	**4.5**	**3.1**	**6.9**	**5.1**	**17.1**
γ-Elemene	34.3	-	-	-	tr	-	-	tr	0.2
E-β-Farnesene	34.9	-	0.4	0.1	0.3	-	-	0.1	0.4
α-Humulene	35.29	0.2	0.7	0.1	0.4	-	-	-	0.9
Aromadendrene	35.57	0.2	0.6	tr	0.3	-	0.1	0.4	0.5
Germacrene D	36.28	-	1.4	0.1	0.6	-	-	0.8	2.3
α-Muurolene	36.79	-	0.8	0.1	0.2	-	-	-	-
ϒ-Cadinene	37.37	0.3	0.6	0.1	0.3	-	0.8	0.6	1.6
δ-Cadinene	37.61	-	-	0.1	0.4	-	0.5	0.6	1.4

Abbreviations of treatment names: Las- = uninfected, Las+ = infected, MJ+ = treated with methyl jasmonate, MJ- = control. Volatiles are listed according to chemical class and within class according to retention time. Tr = compounds that comprise < 0.1% of the total amount of each treatment.

* Values with different letters within each collection day are different at P ≤ 0.0125, planned t-test comparisons.

Compounds marked in bold are responsible for 50% of the differences observed between treatments (SIMPER similarity percentage breakdown procedure).

Forty-six volatile compounds were collected from the four treatment groups, with representatives from seven chemical classes: monoterpenes (23 compounds), sesquiterpenes (10), aliphatic aldehydes (6), green leaf compounds (1), aromatics (2), nitrogen-containing compounds (3), and oxylipins (1) (Table 1). Similarity indices showed that 10 volatile compounds were responsible for 50% of the differences observed in proportional composition between treatments. Of these 10 compounds, similarity of percentages analysis (SIMPER) showed that the monoterpene E-β-ocimene and the aldehyde nonanal, were quantitatively dominant and made the largest contribution to the differences between the treatments (Contribution of E-β-ocimene to (dis)similarity: 9.53%; nonanal: 9.14%). The proportions of these two compounds were in opposition according to treatment: relative to healthy, Tween-treated trees, the proportion of E-β-ocimene was approximately double and comprised over 50% of the total composition in MJ-treated trees, while nonanal comprised a higher proportion in Tween-treated trees and correspondingly diminished in MJ-treated individuals. This effect occurred in collections from both sampling days. The sesquiterpene β-caryophyllene made the third largest contribution to the differences between treatments (6.05%). On day 1 the proportion of β-caryophyllene in Las-infected trees was ca. twice the size as that found in healthy plants; these differences were more pronounced on day 2. Indole was fourth in contribution to the differences between treatments (4.92%). The proportion of indole increased over 100-fold in MJ-treated trees relative to Tween-treated trees on day 1, but on day 2 indole was emitted by only the MJ-treated trees and the amount was diminished relative to day 1. The other compounds that contributed to the remainder of the first 50% of the compositional variation were the aldehydes decanal (4.76%) and octanal (2.84%), the monoterpenes limonene (3.15%), Z-β-ocimene (3.06%), and myrcene (3.03%), and the aromatic methyl salicylate (3.37%). While the oxylipin E-jasmone comprised only a minor proportion of the total volatiles (0.5% on day 1), it was emitted only by MJ-treated trees.

**Fig 2:**
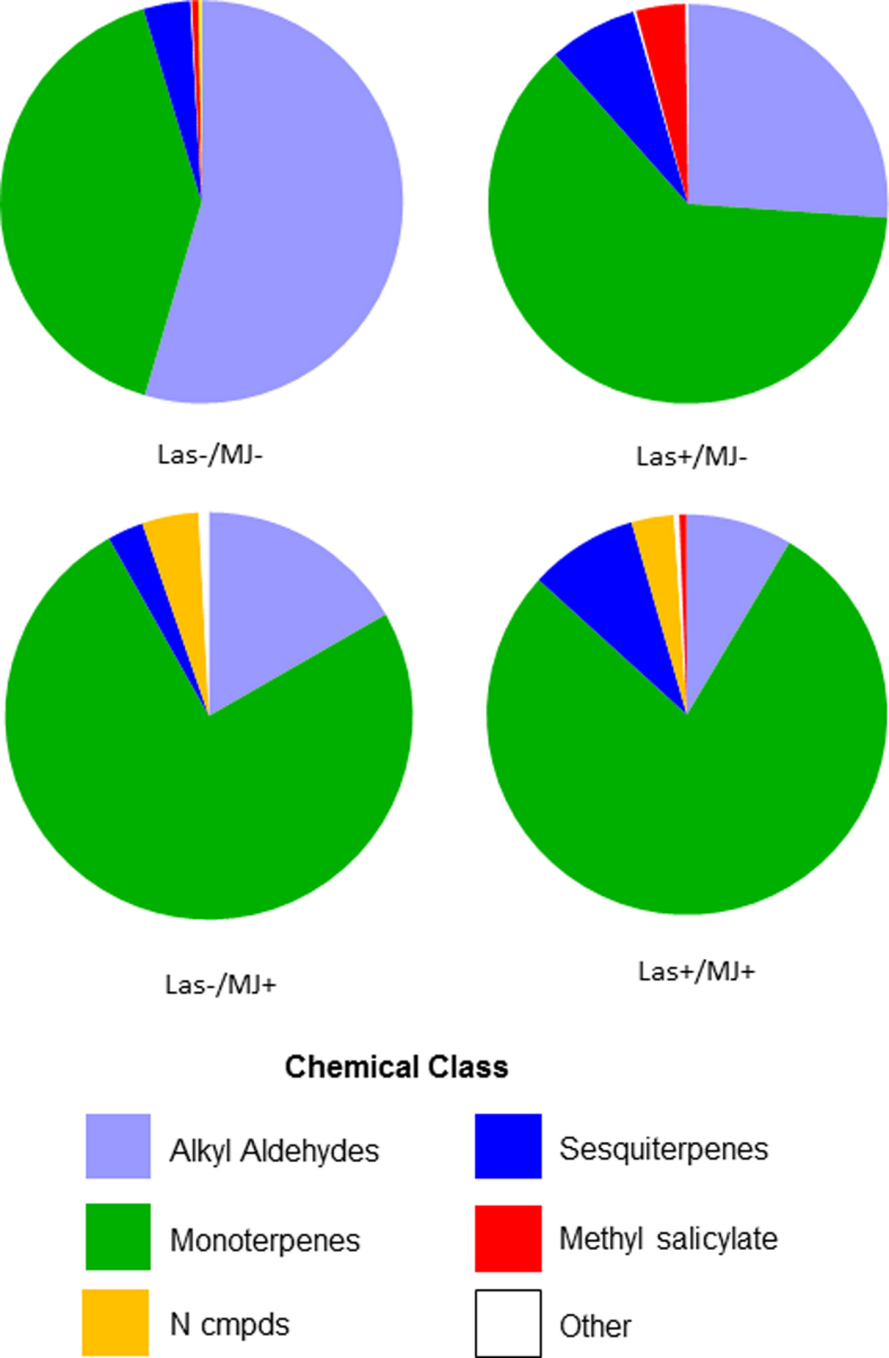
Proportional composition of the individual chemical classes emitted from different treatments in the MJ experiment.

The three main effects, Las infection status, MJ application, and collection day also influenced the proportion of chemical classes observed in the different MJ experiment (S2 Table). There was a weakly significant interaction effect of Las x MJ. The analysis of similarities procedure (ANOSIM) indicated that Las infection and collection day moderately influenced chemical class proportionality (Las infection status: ANOSIM global R = 0.317, *P* ≤ 0.004); collection day: ANOSIM global R = 0.258, *P* ≤ 0.002), whereas MJ treatment had a very strong effect (ANOSIM global R = 0.674, *P* ≤ 0.0001). Similarity of percentages analysis (SIMPER) showed that alkyl aldehydes, monoterpenes, and sesquiterpenes were the main contributors to the observed variation between treatments (alkyl aldehydes: 27.96%; monoterpenes: 22.07%; sesquiterpenes: 20.97%) (Fig 2**)** The proportional changes in nitrogen-based compounds reflected those observed in indole and contributed 15.12%. Methyl salicylate, the primary aromatic compound, was relatively abundant in Tween-treated Las-infected trees, and contributed 9.74% to the observed variation. In terms of total amounts, MJ-treated trees, regardless of Las infection status, emitted ca. 4–6 times more monoterpenes than Tween-treated trees (Fig 2; S3 Table). Most of the increase in amount of monoterpenes was driven by increases in E-β-ocimene, which proportionally increased from 49.2% of the total amount of monoterpenes emitted by uninfected trees to 73% in uninfected MJ-treated trees. Similarly, the proportion of E-β-ocimene increased from 54.2% in Las-infected, Tween-treated trees to 71.4% in Las-infected, MJ-treated trees. The increase in the total amount of nitrogen-containing compounds observed in MJ-treated trees was attributable to the large increase in indole production in these trees.

### Effects of salicylic acid application

**Fig 3:**
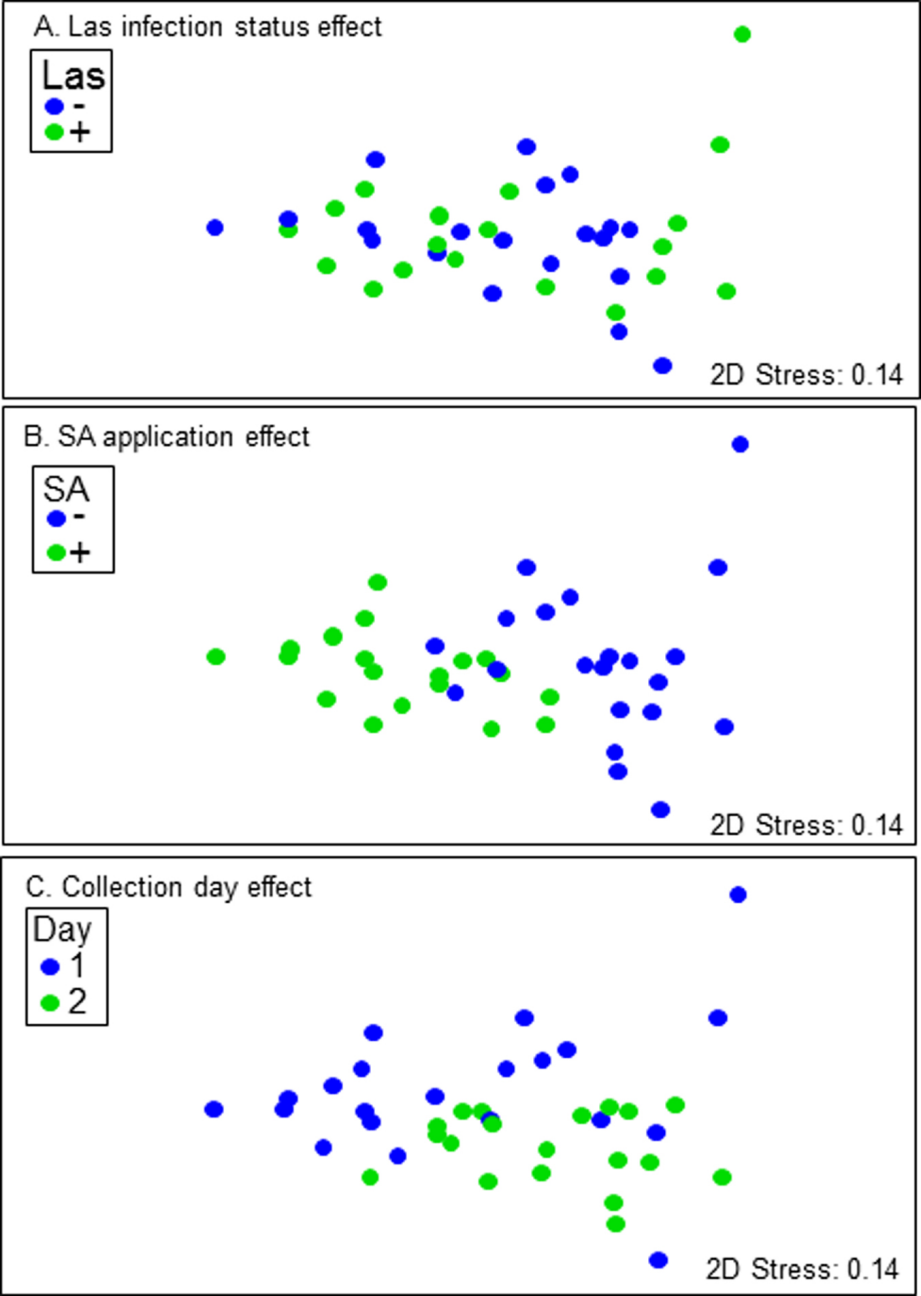
Nonmetric multidimensional scaling (NMDS) of the main effects on proportions of emitted volatile compounds in the SA experiments. NMDS plots showing the effects of (A) Las infection status, (B) SA application, (C) collection day on the proportions of volatiles emitted. Comparisons of the proportions of volatile compounds in each treatment were based on Bray-Curtis similarities plotted in a non-metric multidimensional scaling.

On the first day of sampling, a total of 41 and 44 volatile compounds were collected, respectively, from the foliage of uninfected + Tween-treated and Las-infected + Tween-treated trees (Table 2; S1 Appendix). Fewer volatile compounds, 36 total, were collected from the foliage of SA-treated trees. The total numbers of volatiles emitted in all treatments on day 2 decreased relative to the totals emitted on day 1 (Table 2). Permutational ANOVA showed that two of the main effects, SA application and sampling day, strongly influenced volatile emission; Las infection status, the third main effect, had no influence (S4 Table). The SA application x sampling day interaction effect was significant, but not as strong as the two main effects alone. The analysis of similarities procedure (ANOSIM) also indicated that Las infection status also did not influence volatile composition (ANOSIM global R = -0.082, *P* ≤ 93.6) (Fig 3A), while a stronger effect was observed from SA treatment (ANOSIM global R = 0.537, *P* ≤ 0.0001) (Fig 3B), and a moderate effect from collection day (ANOSIM global R = 0.408, *P* ≤ 0.0001) (Fig 3C).

**Table 2:** Salicylic acid experiment results showing mean absolute amounts, emission rates, and relative proportions of volatile compounds in headspace samples collected on two consecutive days from Valencia sweet orange (infected with Las or uninfected; treated with salicylic acid or Tween-treated).

		DAY 1				DAY 2			
Library/ID	R.T.	Las- SA-	Las+ SA-	Las- SA+	Las+ SA+	Las- SA-	Las+ SA-	Las- SA+	Las+ SA+
No. of compounds		41	44	36	36	33	35	31	33
**Amount collected (ng)**		565	589	3255	2747	291	335	624	677
Emission rate (ng/mg/h)*		0.54a	0.75ab	3.66bc	3.56c	0.26a	0.41ab	0.70b	0.90ab
*Aliphatic aldehydes*									
Heptanal	14.55	1.9	1.2	0.2	-	-	-	-	-
Octanal	18.52	2.9	1.5	0.5	0.6	5.5	3.1	2.8	2.5
**Nonanal**	**22.28**	**13.4**	**12.2**	**3.2**	**3**	**21.5**	**22.4**	**9.6**	**9.7**
**Decanal**	**25.69**	**8.3**	**6.9**	**1.5**	**1.3**	**12.1**	**8.7**	**6.9**	**6.8**
Undecanal	28.92	1.6	1.1	0.3	0.1	0.8	1.2	1.1	0.8
Dodecanal	32.88	0.3	0.3	0.1	0.2	0.3	0.5	0.7	0.8
*Aromatics*									
Benzaldehyde	17.01	0.5	0.4	0.2	0.1	1.1	2.5	0.7	0.5
**Methyl Salicylate**	**25.54**	**1.1**	**11.9**	**69.1**	**67**	**2.1**	**11.9**	**40.6**	**40.5**
*Green leaf volatiles*									
Z-3-Hexenyl acetate	18.63	1.1	0.7	1.1	0.5	-	0.2	0.5	0.4
*Ketones*									
6-methyl-5-Hepten-2-one	17.9	0.4	-	0.7	0.4	-	-	-	-
*Monoterpenes*									
α-Thujene	15.67	0.4	1.1	0.1	0.1	1.3	0.8	-	-
α-Pinene	16	0.8	0.8	0.3	0.3	2.6	1.2	1.3	1
**Sabinene**	**17.53**	**8.3**	**3.9**	**3.2**	**3**	**5.9**	**3.1**	**5.3**	**5.6**
β-Pinene	17.72	1.2	0.8	0.3	0.5	1.5	0.9	1.4	1.3
Myrcene	18.08	2.1	0.6	0.1	1	0.9	-	0.9	0.4
α-Phellandrene	18.72	0.6	1.8	0.3	0.3	2.3	3.6	1.4	0.6
δ-3-Carene	18.96	3.8	1.1	1.7	1.1	2.6	1.5	3.1	2.2
α-Terpinene	19.18	0.5	0.4	0.1	0.2	1.3	0.9	0.6	0.4
**p-Cymene**	**19.48**	**1.9**	**2.6**	**0.3**	**0.5**	**6**	**7.7**	**3.5**	**2.1**
**Limonene**	**19.65**	**5.4**	**2.3**	**3.6**	**1.3**	**4.1**	**4.4**	**4.5**	**4.4**
β-Phellandrene	18.72	-	1.6	-	tr	1.2	2.7	0.8	0.5
Z-β-Ocimene	19.86	0.5	5.8	0.3	0.4	0.4	0.1	0.4	0.4
**E-β-Ocimene**	**20.27**	**25.6**	**11.9**	**7.7**	**13.7**	**13.2**	**8**	**7.8**	**11.8**
ϒ-Terpinene	20.75	0.9	0.7	0.2	0.2	2.6	2.2	1.3	1.1
E-Sabinene hydrate	21.08	1	0.5	0.2	0.3	1.5	1.4	0.8	0.8
Terpinolene	21.85	1.1	0.4	0.4	0.2	1.3	0.4	0.6	0.4
**Linalool**	**22.14**	**2.1**	**2.8**	**1.2**	**1**	**1.5**	**0.5**	**0.2**	**0.6**
allo-Ocimene	23.18	0.5	0.4	0.2	0.3	0.2	-	0.2	0.5
Terpinen-4-ol	24.98	0.3	0.4	0.1	tr	0.2	0.4	0.4	0.3
α-Terpineol	25.46	-	-	-	0.1	-	0.3	-	-
Nerol	26.46	-	0.4	0.1	tr	-	-	-	-
Neral	26.88	0.4	1	0.4	0.2	-	-	-	-
Car-3-en-2-one	27.37	0.3	0.1	tr	tr	0.1	0.1	0.4	0.4
Geranial	27.77	0.7	2.7	0.8	0.4	-	—		-
Geranyl acetone	34.68	1.3	0.5	0.2	0.1	0.4	0.5	0.6	0.4
*N-Compounds*									
Indole	28.71	0.8	1	tr	0.1	-	-	-	0.1
*Sesquiterpenes*									
β-Elemene	32.59	0.8	2.3	0.2	0.1	-	-	-	-
Z-α-Bergamotene	33.47	-	0.4	-	tr	-	0.7	-	0.3
**Caryophyllene**	**33.92**	**4.5**	**10.1**	**0.7**	**1**	**3.9**	**5.5**	**1.5**	**2.7**
E-β-farnesene	34.84	0.4	1.3	tr	tr	0.1	-	-	-
Sesquisabinene	35.01	-	0.1	tr	0.1	-	0.1	-	-
α-Humulene	35.23	0.2	1	tr	-	0.1	0.1	-	-
Germacrene D	36.22	0.3	-	-	-	-	-	-	-
α-Muurolene	36.75	0.2	1.4	tr	0.1	-	-	-	-
β-Bisabolene	36.91	-	0.1	-	tr	-	0.2	-	-
ϒ-Cadinene	37.31	0.9	0.5	0.1	tr	0.7	0.7	0.1	0.1
δ-Cadinene	37.55	0.7	0.6	0.1	0.1	0.7	1.8	0.8	0.3

Abbreviations of treatment names: Las- = uninfected, Las+ = infected, MJ+ = treated with methyl jasmonate, MJ- = control. Volatiles are listed according to chemical class and within class according to retention time. Tr = compounds that comprise < 0.1% of the total amount of each treatment.

* Values with different letters within each collection day are different at P ≤ 0.0125, planned t-test comparisons.

Compounds marked in bold are responsible for 50% of the differences observed between treatments (SIMPER similarity percentage breakdown procedure).

Forty-seven volatile compounds were collected from the four treatment groups, with representatives from seven chemical classes: monoterpenes (25 compounds), sesquiterpenes (11), aliphatic aldehydes (6), green leaf compounds (1), aromatics (2), nitrogen-containing compounds (1), and ketones (1) (Table 2). Similarity indices showed that nine volatile compounds were responsible for 50% of the differences observed in proportional composition between treatments. Methyl salicylate was the quantitatively dominant compound emitted by SA-treated trees and made the largest contribution to the differences between treatments (12.24%); on day 1 it accounted for over 65% of the total composition in both Las- and Las+ trees treated while it accounted for only 1.1% of the total composition in Tween-treated Las- trees. Nonanal and E-β-ocimene were, respectively, the second and fourth largest contributors to differences between treatments (nonanal: 5.33%; E-β-ocimene: 4.38%). In uninfected + Tween-treated trees, E-β-ocimene comprised the largest proportion of volatiles and was nearly twice as abundant as nonanal on day 1; however, in Las-infected + Tween-treated trees, these compounds were emitted in nearly equal proportions. Both compounds comprised a smaller proportion of the total volatiles in the SA-treated trees on day 1. On day 2, the proportional dominance of these two compounds was reversed: In uninfected + Tween-treated trees, the proportion of nonanal was 1.6 times greater than that of E-β-ocimene while in Las-infected + Tween-treated trees, it was 2.8 times greater. Decanal was the third largest contributor to differences between treatments (4.50%). On day 1, the proportion of decanal in uninfected + Tween-treated trees was 5.5 times as great as that in uninfected + SA-treated trees while in Las-infected + Tween-treated trees emitted the proportion of decanal was 4.6 times higher than in Las-infected + SA-treated trees. The differences in decanal proportion between Tween-treated and SA-treated trees were not as pronounced on day 2. The fifth largest contributor to differences between treatments was β-caryophyllene (4.10%). As with decanal, Tween-treated trees had a greater proportion of β-caryophyllene than SA-treated plants; this effect was observed on both collecting days. The other compounds that contributed to the first 50% of the compositional variation were the monoterpenes sabinene (3.68%), p-cymene (3.63%), limonene (3.04%), and linalool (2.86%) and the alkyl aldehyde octanal (3.59%). In contrast to the MJ treatment, SA treatment did not induce emission of either indole or Z-jasmone.

**Fig 4:**
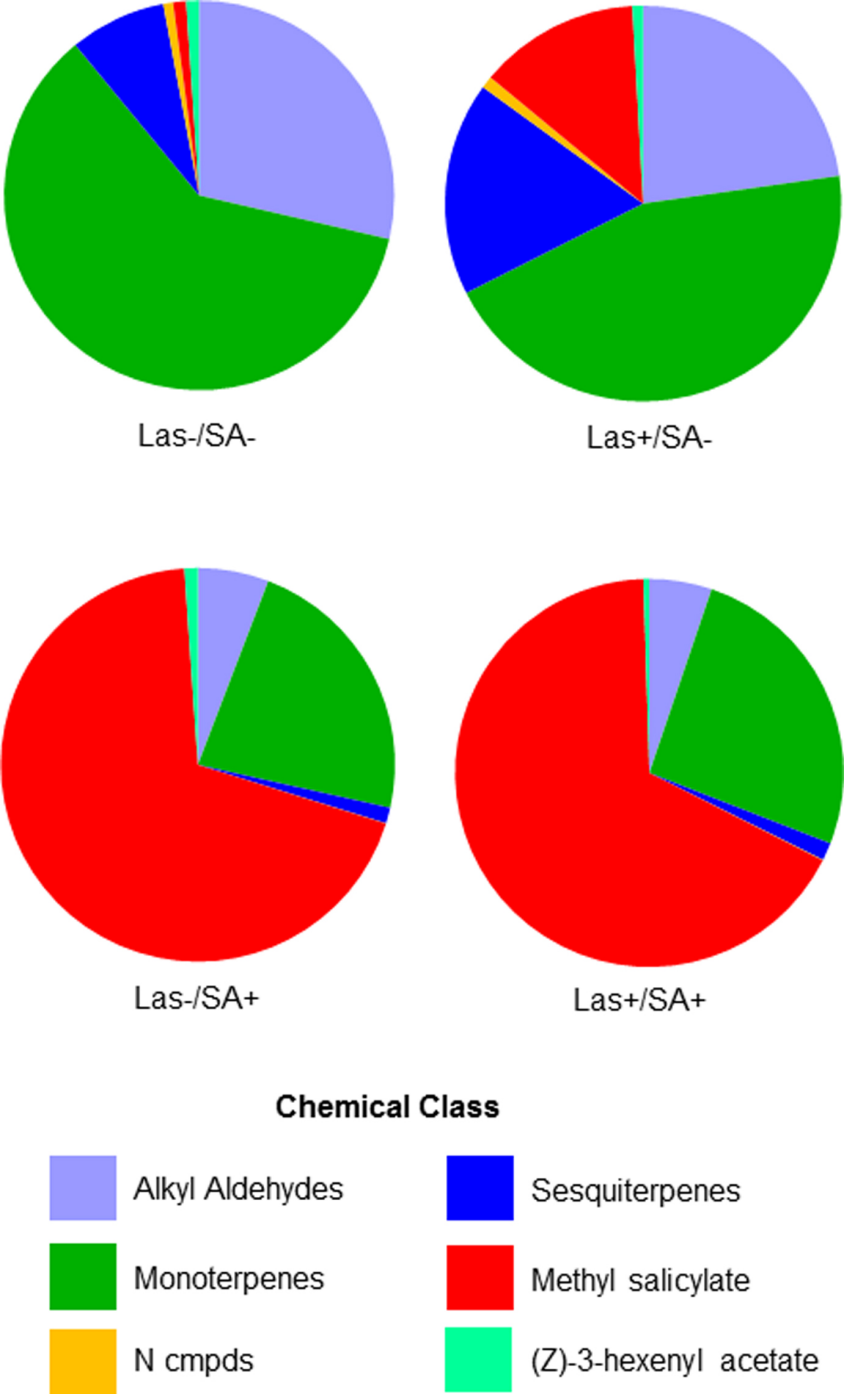
Proportional composition of the individual chemical classes emitted from different treatments in the SA experiment.

Chemical class proportionality in the different SA experimental treatments was also influenced by the three main effects SA application and collection day but not by Las status (S5 Table). There was also a significant interaction effect of SA x Day. The analysis of similarities procedure (ANOSIM) likewise indicated that Las infection did not influence chemical class proportionality (ANOSIM global R = -0.078, NS) while SA treatment had a very strong effect (ANOSIM global R = 0.731, *P* ≤ 0.0001) and collection day had a moderate influence (ANOSIM global R = 0.222, *P* ≤ 0.004). Similarity of percentages analysis (SIMPER) showed that methyl salicylate contributed the most to the (dis)similarity between treatments (42.47%). A relatively large contribution was also made by alkyl aldehydes (20.56%), while moderate contributions were made by monoterpenes (15.16%) and sesquiterpenes (13.75%)(Fig 4; S6 Table) Along with the large amount of methyl salicylate emitted by SA-treated trees, the total amount of monoterpenes emitted by SA-treated trees, regardless of their Las infection status, was ca. 2–3 times higher than Tween-treated trees (Fig 4; S6 Table). E-β-ocimene accounted for some of the increase in the amount of monoterpenes emitted; in uninfected Tween-treated trees on day 1 it comprised 45.4% of the total monoterpene emission while it accounted for 35.0% in Las-infected, Tween-treated trees, 41.0% in uninfected SA-treated trees, and 54.7% in Las-infected, SA-treated trees. On day 2, its proportion decreased across treatments to only ca. 20–30% of the total amount of monoterpenes emitted.

### Psyllid settling behavior

**Fig 5:**
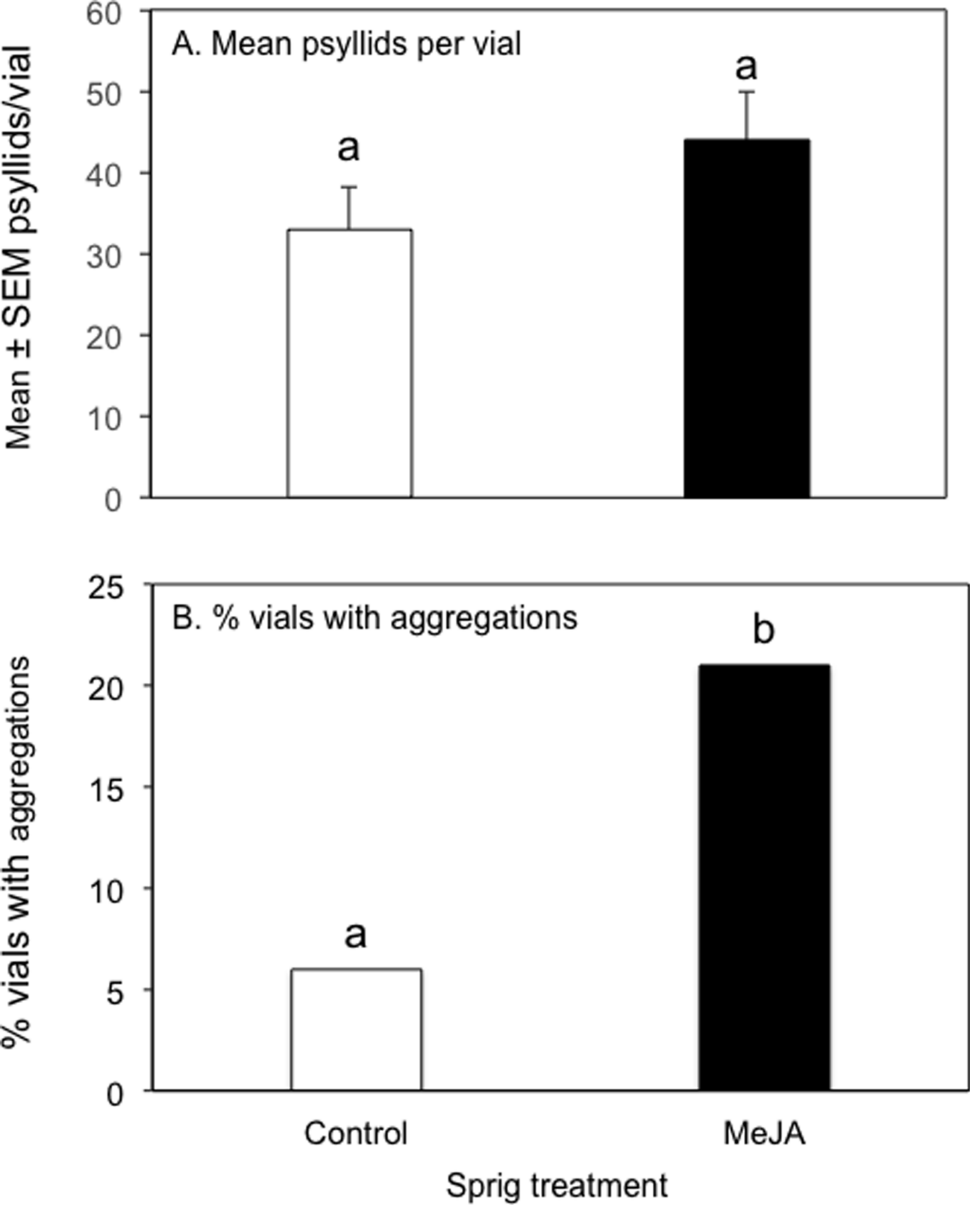
Results of aggregation behavior assay. (A) Mean (± SEM) of psyllids per vial (t = .8928, NS, control n = 8, MJ-treated n = 11); (B) % vials with aggregations (≥ 9 psyllids/vial) (G = 9.48, *P* < 0.01).

There was no significant difference in the mean numbers of adult psyllids that settled on control and MJ-treated sprigs (Control: 33.4 ± 5.1 (mean ± SEM); MJ-treated: 44.0 ± 5.1; *t* = 0.867, *P* = 0.17) (Fig 5A). Similar numbers of males and females were observed in each vial (Control: males: 15.4 ± 3.0, females: 18.0 ± 3.0; *t* = 0.5934, *P* = 0.55; MJ-treated: males: 20.5 ± 2.7, females: 23.5 ± 3.0; *t* = 0.6569, *P* = 0.45). However, psyllids aggregated more frequently on MJ-treated sprigs than on control sprigs (G = 9.48, P ≤ 0.01) (Fig 5B). Of the 81 vials used in the control tests, 6 of them (7.4%) had psyllid aggregations while aggregations were observed in 21 of the 108 vials (19.4%) in the MJ-treatment test.

### Discussion

Exogenous application of both signaler compounds to growing shoots of ‘Valencia’ sweet orange had a pronounced effect on both the emission rate and composition of foliar volatile compounds. However, in our experiments the observed effects appear to be short-lived, with diminishment in emission rates of most compounds observed 24 h after application. This may be because the burst of volatile compounds was energetically-demanding [54]. Longer sampling periods are needed to determine how long the effects on volatile emission from a single application will last. Longer-term observations that track anatomical changes within the leaf, along with changes in the metabolome, transcriptome, and proteome are needed to determine if single applications of MJ or SA have lasting effects on volatile emission or structures associated with it. For example, anatomical changes occurring as a consequence of exogenous signaler application have been observed in other essential oil producing plants, such as conifers, where a single MJ application resulted in increased resin duct production by cambial cells [55]. Examples of induced long-term responses to pathogen attack are also known in *Citrus*. In seedlings of Swingle citromelo (*Citrus paradisi x Poncirus trifoliata*), soil drenches and foliar applications of various SAR inducers induced systemic resistance against citrus canker disease caused by *Xanthomonas citri* and showed season-long control of canker outbreaks on young citrus trees [56]. Maserti et al. [20] found stress- and defense related proteins in clementine (*Citrus* x *clementina*) foliage 72 hours after the placement of spider mites or exogenous MJ application. The findings of these studies suggest that longer-term changes can be induced in *Citrus* by exogenous applications of plant signalers.

Of the volatile compounds that strongly contributed to the differences observed between treatments in the MJ experiment, MeSA and the sesquiterpenes β-caryophyllene were the most indicative of Las infection (Table 1). On day 1, the percent composition of MeSA from Tween-treated + Las-infected trees was six times higher than from the Tween-treated + uninfected trees, while on day 2, the proportion of MeSA from the Tween-treated + Las-infected trees was nine times greater than from the Tween-treated + uninfected trees. This result would be expected since MeSA has been shown to be present in higher levels in Las-infected foliage and is an indicator of Las infection [23, 25–26].

The pattern of MeSA emission from MJ-treated trees was more complicated. The proportion of MeSA from uninfected + MJ-treated trees was 5.7 greater than from Las-infected + MJ-treated trees. MJ application to uninfected trees may have promoted MeSA emission. MJ applied to tomato seeds induced SA production in tomato seedlings [57], and in some plants MeSA emission is influenced by jasmonic acid levels [58]. The lower proportion of MeSA from the Las-infected + MJ-treated trees may have been attributable to Las directly because Las produces an enzyme, salicylate hydroxylase, that degrades salicylic acid (SA) to suppress plant defenses [59].

At the same time, the proportion of MeSA from Las-infected + MJ-treated trees was seven times lower than from Las-infected + Tween-treated trees. This result suggests that MJ application may also reduce the proportion of MeSA emitted by Las-infected trees. If exogenously applied MJ interferes with MeSA emission from Las-infected plants, then MJ application could potentially reduce the attractiveness of Las-infected foliage to *D*. *citri* and help suppress the spread of the disease. This would be especially useful at the initiation of shoot growth when trees are most vulnerable to psyllid colonization and Las transmission [60,61].

β-caryophyllene was also indicative of Las infection in both the MJ and SA experiments; in the SA experiment, β-caryophyllene comprised over 10% of the total volatile emissions from Las-infected + Tween-treated trees (Table 2). Elevated levels of β-caryophyllene and caryophyllene isomers have been previously observed from Las-infected foliage [62,23] and from the foliage of apple trees infected with *Candidatus* Phytoplasma mali, a pathogen vectored by the psyllid *Cacopsylla picta* [34,35]. Hijaz et al. [24] reported elevated levels of β-caryophyllene in ‘Valencia’ sweet orange leaves infested with *D*. *citri*.

In the MJ experiment, Las-infected + uninfected trees emitted larger proportions of several other volatiles, such as Z-3-hexenyl acetate, geranyl acetone, and germacrene D. Similar volatiles, which have antimicrobial activities, are emitted in response to pathogen attack in rough lemon (*Citrus jambhiri*) [19]. Hijaz et al. [63] proposed that these volatile compounds may play a defensive role against Las in *Citrus* because they are both antimicrobials and are abundant in *Citrus* varieties tolerant of Las infection.

In the MJ experiment, an inverse relationship was observed between E-β-ocimene and nonanal, with E-β-ocimene being the quantitatively dominant volatile compound in MJ-treated individuals, regardless of their Las infection status. In uninfected Tween-treated trees, nonanal appeared to comprise a larger proportion of the foliar volatile profile than E-β-ocimene. This was indicated by the relatively higher proportion of nonanal observed in the day 1 from the uninfected + Tween-treated trees and the continued trend towards this state observed in day 2 samples in which nonanal was over 2 times as abundant as E-β-ocimene. The switch to the predominance of E-β-ocimene in the MJ-treated trees is notable because E-β-ocimene has been implicated as an attractant of natural enemies of herbivores [44, 64–66]. E-β-ocimene and its structural isomers Z-β-ocimene and allo-ocimene can also act as a signal molecule for defense-related gene induction [67,68]. For example, exposure of intact *Arabidopsis thaliana* plants to ocimene isomers resulted in substantial changes of the plant transcriptome and induced MJ accumulation [68]. Application of allo-ocimene to *A*. *thaliana* induced defenses against fungal pathogens such as lignification of cell walls and vascular tissue and accumulation of antifungal substances [69].

Small amounts of Z-jasmone were emitted by MJ-treated trees. The induction of this volatile is noteworthy because application of Z-jasmone induced β-ocimene emission in fava beans. Z-jasmone also repels certain aphid species and is an attractant of aphid natural enemies [70]. Z-jasmone can also have interactive effects with methyl salicylate [71]. Possible interactive relationships in *Citrus* between Z-jasmone and ocimene need to be determined.

The fourth largest contributor to differences between treatments in the MJ experiment was indole. Relative to Tween-treated trees, indole increased over 100-fold in MJ-treated trees. Many plant species release indole upon herbivore damage [72]. It is also thought to be a natural enemy attractant [73] and directly decreases food consumption, plant damage and survival of caterpillars of the African cotton leafworm (*Spodoptera littoralis* (Boisduval)) [74]. The gene that expresses indole-3-glycerol phosphate lyase, an enzyme that helps form indole, is induced by herbivory, the insect-derived salivary elicitor volicitin, and MJ treatment [75,76].

Since ocimene isomers, Z-jasmonate, and indole can prime certain plant species for impending attack by herbivores [68,70,73], it would be useful to determine whether *Citrus* can be primed for herbivore attack by these or other volatile compounds. Consecutive, shorter interval sampling periods for volatile compounds following the application of signaler compounds would be a necessary first step for determining whether potential primer volatiles like indole or ocimene isomers are emitted prior to the onset of other volatiles.

Methyl salicylate emission erupted in SA-treated plants, regardless of their Las infection status, and comprised > 65% of total volatile emitted on day 1. Since volatile production is energetically-demanding [54], it is interesting that monoterpene output also roughly doubled following treatment with SA (S6 Table). SA application had little effect on the proportions of sesquiterpenes and alkyl aldehyde that were emitted. The proportionality shifts of E-β-ocimene and nonanal were not as evident in the SA experiment as in the MJ experiment. Nor was E-β-ocimene as proportionally dominant in the SA-treated trees as it was in the MJ-treated trees. Of the other compounds that contributed to the first 50% of the (dis)similarity observed between treatments in the SA experiment, sabinene, p-cymene, limonene, linalool, and β-caryophyllene showed decreased proportions in the SA-treated trees on day 1. This effect began to diminish on day 2 when the proportion of methyl salicylate also decreased to ca. 40% of the total volatiles compounds emitted.

The behavioral assay indicated that MJ application and the induced changes it causes in volatile emission influenced host plant selection behavior of *D*. *citri*. While there was no significant difference in the mean numbers of psyllids that settled on MJ-treated versus Tween-treated sprigs, it is noteworthy that a significantly greater percentage of the MJ-treated sprigs had aggregations of nine or more psyllids on them. Adult *D*. *citri* aggregate on flushing shoots of their host plants [77,78]. Further work is needed to determine whether the enhanced aggregation response was due to increased emission levels, changes in volatile composition, or a combination of both factors arising from the MJ-treated sprigs. Another possibility is that on day 2 of the volatile study, the proportion of MeSA comprising the volatiles emitted by the uninfected + MJ-treated trees was seven times greater than that emitted by uninfected + Tween-treated trees. The increasing proportion of methyl salicylate in uninfected + MJ-treated sprigs used in the behavioral assay may have played a role in inducing aggregation behavior in the psyllids. Increased levels of MeSA are associated with *D*. *citri* attraction to Las-infected citrus shoots [23,26,28]. The proportional changes observed in the 10 compounds contributing to 50% of the (dis)similarity between treatments may provide a template for further studies to determine the identities of volatiles that most strongly influence *D*. *citri* aggregation behavior.

Induced resistance in *Citrus* is of current interest because of the threat posed by huanglongbing disease to commercial citrus worldwide. The results of our study show that exogenous application of MJ and SA has immediate and significant effects on the composition and emission levels of foliar volatiles from *Citrus*. Further studies are needed to determine whether application of these compounds has the potential to be used to help manage the disease in commercial citrus (See Li et al. [79]). The effects seen here were short-lived but longer-lasting effects may have been induced and will require more extensive investigation. The dynamics between inducible defense pathways and signaling agents is complex [80] and is further complicated by the influence of the pathogen itself. Studies can be directed toward determining whether MJ application interferes with MeSA emission from Las-infected trees, thus reducing *D*. *citri* recruitment. It may also be possible to use MJ application as a means of increasing recruitment of natural enemies into areas infested with *D*. *citri* resulting in a reduction in their local population levels.

## Supporting information

S1 Appendix Percent composition of volatiles emitted by flushing shoots of Valencia sweet orange in each treatment. Las- = uninfected; Las+ = infected with *Candidatus* Liberibacter asiaticus; MJ+ = sprayed with methyl jasmonate; MJ- = control spray; SA+ = sprayed with salicylic acid; SA- = control spray.
(PDF)

S1 Table Permutational MANOVA analysis of the effects of Las infection status, methyl jasmonate application, and collection day (Day 1 v. Day 2) on the proportions of volatile compounds emitted from Valencia sweet orange. Data were square root transformed prior to analysis.
(PDF)

S2 Table Permutational MANOVA analysis of the effects of Las infection status, methyl jasmonate application, and collection day (Day 1 v. Day 2) on the proportions of chemical classes emitted from Valencia sweet orange. Data were square root transformed prior to analysis.
(PDF)

S3 Table Total amount (ng) of each chemical class and selected compounds emitted in the MJ Experiment (n = 5 replicates per treatment). AA: alkyl aldehydes, MT: monoterpenes, ST: sesquiterpenes, N: Nitrogen-compounds, E-J: Z- jasmone, MeJA: Methyl salicylate.
(PDF)

S4 Table Permutational MANOVA analysis of the effects of Las infection status, salicylic acid application, and collection day (Day 1 v. Day 2) on the proportions of volatile compounds emitted from Valencia sweet orange. Data were square root transformed prior to analysis.
(PDF)

S5 Table Permutational MANOVA analysis of the effects of Las infection status, salicylic acid application, and collection day (Day 1 v. Day 2) on the proportions of chemical classes emitted from Valencia sweet orange. Data were square root transformed prior to analysis.
(PDF)

S6 Table Total amount (ng) of each chemical class and selected compounds emitted in the SA Experiment (n = 5 replicates per treatment). AA: alkyl aldehydes, MT: monoterpenes, ST: sesquiterpenes, N: Nitrogen-compounds, MeJA: Methyl salicylate.
(PDF)
