# Supplementary material for: Exogenous application of the plant signalers methyl jasmonate and salicylic acid induces changes in volatile emissions from citrus foliage and influences the aggregation behavior of Asian citrus psyllid (Diaphorina citri), vector of Huanglongbing
Source: PLoS One. 2018 Mar 29;13(3):e0193724. doi: 10.1371/journal.pone.0193724 (PMC5875780; doi:10.1371/journal.pone.0193724)
Supplement: S1 Appendix — Las- = uninfected; Las+ = infected with Candidatus Liberibacter asiaticus; MJ+ = sprayed with methyl jasmonate; MJ- = control spray; SA+ = sprayed with salicylic acid; SA- = control spray. (PDF) [file pone.0193724.s001.pdf]

Las-/MJ-/ Day 1

		% Composition/Collection Date					
R.T.	Library/ID	Aug12-13	Aug19-20	Oct 21-22	Nov 4-5	Nov 22-23	Mean
14.58	Heptanal	2.1	2.5	0	0	0.0	0.9
16.03	Pinene<alpha->	1.0	0.5	2.1	2.0	0.9	1.3
17.57	Sabinene	6.2	3.8	0	1.9	5.9	3.6
17.75	Pinene<beta->	1.4	1.3	0	0	1.9	0.9
18.12	Myrcene	2.4	2.3	0	0	3.3	1.6
18.56	Octanal	1.8	4.2	2.4	5.0	3.9	3.4
18.67	Z-3-hexenyl acetate	0.5	0	0	0	0	0.1
18.76	.alpha.-Phellandrene	0.9	0	0	0	0	0.2
19.00	Carene<delta-3->	2.9	1.3	0	1.6	0	1.2
19.21	α-Terpinene	0	0	0	0	0.4	0.1
19.52	Cymene<para->	0.5	1.0	0	0	0	0.3
19.69	Limonene	6.3	2.3	5.2	1.5	2.2	3.5
19.80	Benzyl Alcohol	0.4	0.5	0	0	0	0.2
19.89	Ocimene<(Z)-beta->	1.4	0.9	0	0.6	1.1	0.8
20.31	Ocimene<(E)-beta->	46.9	23.4	19.1	8.5	33.3	26.2
20.80	Terpinene<gamma->	0.7	1.1	0	0	0.7	0.5
21.12	Sabinene hydrate	0.7	0.5	0	0	0	0.2
21.90	Terpinolene	0.7	0.7	0	0	0.7	0.4
22.17	Linalool	0	2.3	0	0	0	0.5
22.32	Nonanal<n->	19.5	15.6	68.0	47.1	27.3	35.5
23.22	Ocimene<allo->	0.9	0.4	0	0	1.0	0.5
23.31	1,3,8-p-Menthatriene	0	0.7	0	0	0	0.1
25.02	4-Terpinol	0	0.7	0	0	0	0.1
25.58	Methyl Salicylate	0.3	0.9	0.7	0.5	0.5	0.6
25.72	15.83 Decanal<n->	2.0	10.7	2.6	21.4	12.8	9.9
28.75	Indole	0.5	0.1	0	0	0.0	0.1
28.96	Undecanal	0.02	1.3	0	6.0	1.6	1.8
32.94	Dodecanal	0.003	0	0	1.6	0.9	0.5
33.98	Caryophyllene(E-)	0.2	12.7	0	0	0.8	2.8
35.29	Humulene<alpha->	0	0.8	0	0	0.0	0.2
35.56	Aromadendrene<allo->	0	0.9	0	0	0	0.2
37.37	Cadinene<gamma->	0	1.3	0	0	0	0.3
37.60	(+)-δ-Cadinene	0	1.4	0	0	0	0.3

Las-/MJ+/DAY 1

% Composition/Collection Date

R.T.	Library/ID	Aug12-13	Aug19-20	Oct 21-22	Nov 4-5	Nov 22-23	Mean
16.02	Pinene<alpha->	0.3	0.4	0.5	0.8	0.4	0.5
17.57	Sabinene	1.0	3.2	1.5	3.4	6.0	3.0
17.75	beta.-Pinene	0.9	0	1.0	2.0	0.5	0.9
18.13	Myrcene	2.3	2.1	2.1	3.5	2.4	2.5
18.56	Octanal	0.9	0.8	1.3	3.0	0.7	1.3
18.67	Hexenyl acetate<3E->	0	0	0	0	0.3	0.1
19.00	Carene<delta-3->	0.3	1.2	0.5	1.5	1.6	1.0
19.22	α-terpinene	0.1	0	0	0	0.2	0.1
19.50	Cymene<ortho->	0	0.1	0.3	1.1	0.2	0.4
19.69	Limonene	0.3	2.8	0.6	2.2	1.5	1.5
19.79	Benzyl alcohol	0.4	0	0	0	0	0.1
19.90	Ocimene<(Z)-beta->	2.2	2.0	1.5	1.4	2.1	1.8
20.35	Ocimene<(E)-beta->	65.5	72.7	53.0	45.8	62.2	59.8
20.81	Terpinene<gamma->	0.4	0.2	0.4	0.8	0.3	0.4
21.12	Sabinene hydrate<trans->	0.3	0.3	0.5	0.7	0.3	0.4
21.90	Terpinolene	0.2	0.2	0.1	0	0.4	0.2
22.17	Linalool	0	1.3	0	0	1.8	0.6
22.33	Nonanal<n->	10.4	3.4	20.3	15.7	3.7	10.7
23.22	Ocimene<allo->	1.3	1.1	1.1	1.1	1.7	1.2
23.31	Menthatriene<1,3,8-para->	0	0	0	0	0.7	0.1
23.66	Ocimene<allo->	0	0	0	0	0.3	0.1
25.58	Methyl salicylate	0.4	0	0.4	0.3	0.2	0.3
25.73	Decanal<n->	1.8	1.4	5.6	8.8	1.3	3.8
27.80	Geranial	0	0.3	0	0	0.5	0.2
28.74	Indole	8.4	4.0	3.9	3.1	3.1	4.5
28.97	Undecanal	0.2	0	0.5	2.1	0.3	0.6
32.82	Jasmone<E->	0.7	0.5	0.5	0.7	0.1	0.5
32.94	Dodecanal	0	0	0	0	0	0.3
33.98	Caryophyllene(E-)	1.0	0.8	3.3	1.1	3.6	2.0
34.73	Geranyl acetone	0	0	0.5	0	0	0.1
34.89	Farnesene<(E)-beta->	0	0	0	0	0.4	0.1
35.28	Humulene<alpha->	0	0	0.1	0	0.3	0.1
35.55	Aromadendrene	0	0	0	0	0.1	0.03
36.28	Germacrene D	0.5	0	0	0	0.1	0.1
36.78	α-Muurolene	0	0	0	0	0.5	0.1
37.37	Cadinene<gamma->	0.1	0.1	0.2	0	0.1	0.1
37.60	Cadinene<delta->	0.1	0	0.2	0	0.2	0.1

Las+/MJ-/DAY 1

R.T.	Library/ID	% Composition/Collection Date					
		Aug12-13	Aug19-20	Oct 21-22	Nov 4-5	Nov 22-23	Mean
16.02	Pinene<alpha->	1.0	0.7	1.2	0.5	0.6	0.8
17.57	Sabinene	3.9	5.8	17.8	2.7	3.3	6.7
17.75	beta.-Pinene	1.9	1.0	2.0	1.0	1.4	1.5
18.12	beta.-Myrcene	3.0	2.4	3.4	2.3	1.6	2.5
18.56	Octanal	0.7	1.5	1.6	2.0	5.0	2.2
18.67	Hexenyl acetate<3Z->	0	1.1	0	2.0	0	0.6
18.99	Carene<delta-3->	1.6	2.6	4.3	0.8	0.4	1.9
19.22	α-Terpinene	0.3	0	0.4	0.4	1.3	0.5
19.51	Cymene<para->	1.3	1.0	0.9	0.9	0.8	1.0
19.68	Limonene	2.2	2.9	12.3	0.7	0	3.6
19.79	Benzyl alcohol	0.6	0.4	0	0	0	0.2
19.89	Ocimene<(Z)-beta->	1.3	1.6	0.6	1.6	0.8	1.2
20.31	Ocimene<(E)-beta->	40.7	46.8	21.8	49.7	24.8	36.8
20.79	Terpinene<gamma->	1.1	0.8	0.7	0.8	0.9	0.9
21.12	Sabinene hydrate<trans->	0.6	0.5	0.8	0.4	0	0.5
21.89	Terpinolene	0.6	0.7	1.2	0	0.6	0.6
22.17	Linalool	0	2.4	5.8	0	0	1.6
22.32	Nonanal<n->	26.5	7.4	14.5	11.0	25.5	17.0
23.22	Ocimene<allo->	0.9	1.1	0.5	1.2	0.5	0.8
23.66	Ocimene<neo-allo->	0	0	0	0.2	0	0.05
25.58	Methyl Salicylate	3.9	2.5	0.6	9.9	3.3	4.0
25.72	Decanal<n->	5.8	4.3	0	5.0	10.5	5.1
28.75	Indole	0.1	0.5	0	0.4	0	0.2
28.96	Undecanal	0.3		0.4	1.1	1.4	0.8
32.64	Elemene<beta->	0	0	1.3	0.3	0	0.3
32.94	Dodecanal	0.2	0.3	0	0.5	0.6	0.3
33.98	Caryophyllene(E-)	0.4	3.4	1.8	3.5	14.3	4.7
34.73	Geranyl acetone	0	0.5	0	0	0.7	0.2
34.89	Farnesene<(E)-beta->	0	0.4	0.5	0	0	0.2
35.28	Humulene<alpha->	0	0.5	0	0	0.9	0.3
35.56	Aromadendrene<allo->	0	1.0	0	0.3	0	0.3
36.27	Germacrene D	0	1.4	0	0	0	0.3
36.79	α-Muurolene	0	0.9	0.6	0.0	0	0.3
37.38	Cadinene<gamma->	0.5	0.9	0	0.4	0.6	0.5
37.61	Cadinene<delta->	0.6	0.9	0.2	0.3	0	0.4

Las+/MJ+/DAY 1

		% Composition/Collection Date					
R.T.	Library/ID	Aug12-13	Aug19-20	Oct 21-22	Nov 4-5	Nov 22-23	Mean
12.74	Butyl aldoxime, 3-methyl-, syn-	0.2	0	0	0	0	0.04
12.82	Butyl aldoxime, 2-methyl-, syn-	0.2	0	0	0	0	0.04
13.41	Butyl aldoxime, 3-methyl-, anti	0.2	0	0	0	0	0.04
16.03	Pinene<alpha->	0.7	0.3	0.7	0.3	0.3	0.5
17.58	Sabinene	7.0	3.0	8.2	1.2	1.0	4.1
17.76	Pinene<beta->	0.9	0.4	0.9	0.9	0	0.6
18.13	Myrcene	2.6	2.1	2.5	0	0	1.4
18.56	Octanal	0.7	0.4	0.5	0	1.1	0.5
18.68	Hexenyl acetate<3Z->	0.4	0.7	0	0	0	0.2
18.77	Phellandrene<alpha->	0.4	0	0	0	0.5	0.2
19.00	Carene<delta-3->	1.9	1.3	3.8	0.5	0.3	1.6
19.23	α-terpinene	0.4	0.2	0.3	0.4	0.2	0.3
19.52	Cymene<ortho->	0.6	0.2	0.2	0.5	0.3	0.4
19.69	Limonene	3.5	1.2	6.9	0.4	0.5	2.5
19.80	Benzyl alcohol	0.3	0.1	0	0	0	0.1
19.90	Ocimene<(Z)-beta->	1.8	2.4	1.4	2.1	2.2	2.0
20.37	Ocimene<(E)-beta->	56.3	64.7	48.0	59.6	66.9	59.1
20.81	Terpinene<gamma->	0.7	0.4	0.4	0.5	0.3	0.4
21.12	Sabinene hydrate<trans->	0.5	0.4	0.7	0.5	0.3	0.5
21.90	Terpinolene	0.5	0.4	0.8	0.5	0.1	0.5
22.17	Linalool	0	1.0	3.0	0	0	0.8
22.32	Nonanal<n->	4.8	2.4	4.7	5.8	9.4	5.4
23.22	Ocimene<allo->	1.4	0	1.1	1.4	1.8	1.1
23.31	Menthatriene<1,3,8-para->	0	0.6	0	0	0	0.1
25.02	Terpinen-4-ol	0.5	0	0	0.5	0	0.2
25.42	(+)-.alpha.-Terpineol	0	0.2	0.6	0	0	0.2
25.58	Methyl Salicylate	0.3	0.2	0.2	0.3	1.6	0.5
25.73	Decanal<n->	2.6	0.9	1.6	2.7	2.7	2.1
26.91	Neral	0	0.3	0.7	0	0	0.2
27.42	Car-3-en-2-one	0.2	0	0	0.3	0	0.1
27.81	Geranial	0.8	0.5	1.2	0	0	0.5
28.74	Indole	4.3	3.0	1.9	3.5	4.1	3.4
28.97	Undecanal	0.3	0.2	0.2	0.7	0.5	0.4
32.65	Elemene<beta->	0	0.3	1.5	0.3	0	0.4
32.82	Jasmone<E->	0.4	0.6	0.3	1.0	0.3	0.5
32.94	Dodecanal	0.2	0	0.1	0.3	0.2	0.1
33.98	Caryophyllene(E-)	2.2	0	3.9	12.1	4.0	4.5
34.30	γ-elemene	0.2	0	0	0	0	0.0
34.73	Geranyl acetone		0.2	0.2	0	0.3	0.1
34.90	Farnesene<(E)-beta->	0.3	0.2	0.5	0.3	0.1	0.3
35.29	Humulene<alpha->	0	0.5	0.5	0.8	0.3	0.4
35.57	Aromadendrene	0.4	0.3	0.2	0.5	0.2	0.3
36.28	Germacrene D	0.9	0.6	0.5	0.7	0	0.6
36.79	α-Muurolene	0	0.3	0.6	0	0	0.2
37.37	Cadinene<gamma->	0.4	0.3	0.1	0.7	0.2	0.3
37.61	Cadinene<delta->	0.5	0.3	0.2	0.7	0.2	0.4

Las-/MJ-/DAY 2

% Composition/ Collection Date

R.T.	Library/ID	Aug12-13	Aug19-20	Oct 21-22	Nov 4-5	Nov 22-23	Mean
14.58	Heptanal	0.0	3.3	0	0	0.0	0.7
16.03	Pinene<alpha->	2.1	0.7	45.0	2.7	1.0	10.3
17.57	Sabinene	2.6	3.9	0	4.0	2.0	2.5
18.56	Octanal	6.8	7.1	0	6.2	4.2	4.9
19.00	Carene<delta-3->	1.7	1.4	0	2.4	0	1.1
19.21	α-Terpinene	0	0.9	0	0	0	0.2
19.52	Cymene<para->	2.3	3.5	0	5.1	3.6	2.9
19.69	Limonene	2.4	2.0	0	0	1.8	1.2
19.89	Ocimene<(Z)-beta->	0.9	0.4	0	0	0	0.3
20.31	Ocimene<(E)-beta->	21.3	6.4	17.9	17.3	12.0	15.0
20.80	Terpinene<gamma->	1.1	1.9	0	0	0	0.6
21.90	Terpinolene	0	1.0	0	0	0	0.2
22.32	Nonanal<n->	39.4	27.1	0	41.9	56.7	33.0
25.02	4-Terpinol	0	1.1	0	0	0	0.2
25.58	Methyl Salicylate	0.4	1.5	0	1.6	1.0	0.9
25.72	Decanal<n->	17.6	17.7	28.1	16.0	12.0	18.3
28.96	Undecanal	1.4	3.7	0	1.9	4.9	2.4
32.94	Dodecanal	0	0.8	9.3	1.0	0	2.2
33.98	Caryophyllene(E-)	0	15.6	0	0	0	3.1

Las-/MJ+/DAY 2

		% Composition/Collection Date					
R.T.	Library/ID	Aug12-13	Aug19-20	Oct 21-22	Nov 4-5	Nov 22-23	Mean
16.02	Pinene<alpha->	0.7	0.8	1.1	2.0	0.6	1.0
17.57	Sabinene	1.4	2.0	2.7	2.6	4.8	2.7
17.75	beta.-Pinene	0.9	1.2	2.0	5.6	1.2	2.2
18.13	Myrcene	2.0	0	0	0	2.4	0.9
18.56	Octanal	1.0	1.7	2.9	5.3	1.4	2.5
18.67	Hexenyl acetate<3E->	0	0	1.0	0	0	0.2
18.75	α-phellandrene	0	0	0.8	0	1.9	0.5
19.00	Carene<delta-3->	0.6	1.3	0.9	2.1	2.1	1.4
19.22	α-Terpinene	0.2	0	0.6	0	0.5	0.3
19.50	Cymene<ortho->	0.5	0.6	0	2.5	1.0	0.9
19.69	Limonene	0.5	0.8	0.6	0	3.6	1.1
19.73	Phellandrene<beta->	0	0	2.8	1.9	0	0.9
19.90	Ocimene<(Z)-beta->	2.0	2.0	0.8	35.2	1.4	8.3
20.35	Ocimene<(E)-beta->	64.3	67.0	25.0	1.5	47.6	41.1
20.81	Terpinene<gamma->	0.5	0.6	1.0	0	0.6	0.5
21.12	Sabinene hydrate<trans->	0.3	0.5	0.7	0	0.5	0.4
21.90	Terpinolene	0.3	0	0.7	0	0.7	0.3
22.17	Linalool	0	0	0	0	1.2	0.2
22.33	Nonanal<n->	10.0	6.5	29.1	19.2	11.6	15.3
23.22	Ocimene<allo->	1.2	1.4	0.7	1.1	0.9	1.0
28.74	Benzyl cyanide	0.5	0.6	0	0	0	0.2
25.58	Methyl salicylate	1.7	0.5	1.2	2.1	0.5	1.2
25.73	Decanal<n->	2.7	4.2	8.4	15.5	3.2	6.8
27.42	Car-3-en-2-one	0	0	0.2	0	0	0.0
28.74	Indole	1.8	0.6	1.2	0	0.9	0.9
28.97	Undecanal	0.4	0.8	0.8	2.4	0.5	1.0
32.82	Jasmone<E->	0	0.2	0	0	0	0.0
32.94	Dodecanal	0.1	0	0	1.1	0	0.2
33.98	Caryophyllene(E-)	3.0	2.5	11.4	0	8.5	5.1
34.30	γ-Elemene	0.2	0	0	0	0	0.0
34.73	Geranyl acetone	0.7	0	0	0	0	0.2
34.89	Farnesene<(E)-beta->	0	0.3	0	0	0.3	0.1
35.55	Aromadendrene<allo->	0.3	0.7	0.8	0	0	0.38
36.28	Germacrene D	1.4	1.1	0	0	1.6	0.8
37.37	Cadinene<gamma->	0.4	0.8	1.2	0	0.4	0.6
37.60	Cadinene<delta->	0.4	1.3	1.4	0	0	0.6

Las+/MJ-/DAY 2

R.T.	Library/ID	% Composition/ Collection Date Aug12-13	Aug19-20	Oct 21-22	Nov 4-5	Nov 22-23	Mean
16.02	Pinene<alpha->	2.1	1.1	0.9	1.8	1.2	1.4
17.57	Sabinene	6.1	5.3	3.4	3.7	2.6	4.2
17.75	.beta.-Pinene	3.2	1.9	2.3	2.2	0	1.9
18.12	.beta.-Myrcene	0	0	2.8	0	0	0.6
18.56	Octanal	2.3	3.5	4.1	3.2	1.4	2.9
18.76	.alpha.-Phellandrene	0	0	1.4	0	0	0.3
18.99	Carene<delta-3->	2.8	1.5	1.2	0	0	1.1
19.22	α-Terpinene	2.1	0.5	0.7	1.4	0	0.9
19.51	Cymene<para->	4.8	2.2	1.7	4.2	0	2.6
19.68	Limonene	2.1	1.7	6.2	2.2	0	2.4
19.89	Ocimene<(Z)-beta->	0.9	0.7	0.9	0	0	0.5
20.31	Ocimene<(E)-beta->	32.7	17.6	22.2	20.4	13.7	21.3
20.79	Terpinene<gamma->	4.9	2.0	1.5	2.2	0	2.1
21.12	Sabinene hydrate	1.5	0.8	0.7	0	0	0.6
21.89	Terpinolene	1.8	0.9	0.9	0	0	0.7
22.32	Nonanal<n->	24.2	26.5	30.6	7.2	39.5	25.6
23.22	Ocimene<allo->	0.7	0.5	0.5	0	0	0.3
25.02	14.66 Terpinen-4-ol	0	1.2	0	0	0	0.23
25.58	Methyl Salicylate	1.2	16.6	4.4	7.9	10.7	8.1
25.72	Decanal<n->	0.7	10.1	9.1	8.6	4.9	6.7
28.96	Undecanal	0.1	1.4	0.8	2.2	1.7	1.2
32.94	Dodecanal	0	0	0	0.6	0	0.1
33.98	Caryophyllene(E-)	3.8	1.6	2.3	5.7	21.2	6.9
34.73	Geranyl acetone	0	1.2	0.6	1.3	0	0.6
35.56	Aromadendrene<allo->	0	0.7	0	0	0	0.1
37.38	Cadinene<gamma->	1.2	0.8	0.7	1.1	0	0.8
37.61	Cadinene<delta->	1.0	0	0	1.3	0	0.5

Las+/MJ+/DAY 2

		% Composition/ Collection Dates					
R.T.	Library/ID	Aug12-13	Aug19-20	Oct 21-22	Nov 4-5	Nov 22-23	Mean
12.74	Butyl aldoxime, 3-methyl-, syn-	0	0.5	0	0	0	0.11
12.82	Butyl aldoxime, 2-methyl-, syn-	0	0.3	0	0	0	0.07
16.03	Pinene<alpha->	0.7	0.8	0.7	0.6	0.6	0.7
17.58	Sabinene	2.8	1.7	1.9	2.1	1.9	2.1
17.76	Pinene<beta->	1.3	1.2	1.4	1.6	1.4	1.4
18.13	Myrcene	1.9	1.8	1.9	2.3	2.2	2.0
18.56	Octanal	0.5	2.0	2.3	1.2	2.0	1.6
18.68	Hexenyl acetate<3Z->	0	0.8	0	1.0	0	0.4
19.00	Carene<delta-3->	1.2	0.9	0.7	0.9	0	0.7
19.23	α-terpinene	0.7	0.3	0.4	0	0	0.3
19.52	Cymene<ortho->	1.0	0.7	0.6	0.9	1.7	1.0
19.69	Limonene	1.3	0.5	0.4	0.6	0.9	0.8
19.73	Phellandrene<beta->	0	0	1.7	0	0	0.3
19.90	Ocimene<(Z)-beta->	1.8	1.3	0	0.7	1.2	1.0
20.37	Ocimene<(E)-beta->	53.6	40.6	0	18.9	33.0	29.2
20.81	Terpinene<gamma->	1.3	1.0	0	1.1	0.8	0.8
21.12	Sabinene hydrate<trans	0	0.6	0	0.6	0	0.3
21.90	Terpinolene	0.6	0.4	0	0.6	0	0.3
22.32	Nonanal<n->	4.2	10.2	14.1	8.1	22.2	11.8
23.22	Ocimene<allo->	1.0	0.9	0.9	0.5	0.9	0.8
23.64	Benzyl cyanide	0.3	0.7	0	0	0.6	0.3
25.02	14.66 Terpinen-4-ol	0	0.6	0	0	0	0.1
25.42	alpha.-Terpineol	0	0	0.4	0	0	0.1
25.58	Methyl Salicylate	1.0	1.2	0.5	0.4	3.0	1.2
27.42	Car-3-en-2-one	0	0	0	0.4	0	0.1
28.74	Indole	2.1	3.3	1.1	1.7	1.6	2.0
28.97	Undecanal	0.1	1.0	0.2	0.5	1.6	0.7
32.82	Jasmone<E->	0	0.2	0	0	0	0.0
32.94	Dodecanal	0	0.5	0.1	0.1	0.4	0.2
33.54	25.99 Bergamotene<alpha-tran	0.2	0	0	0.4	0	0.1
33.98	25.36 Caryophyllene(E-)	12.8	11.4	12.0	35.5	13.9	17.1
34.30	(-)-γ-elemene	0	0	0.4	0.3	0	0.2
34.73	26.85 Geranyl acetone	0	0	0	0	0.7	0.1
34.90	26.92 Farnesene<(E)-beta->	0	0.4	0.6	0.4	0.4	0.4
35.29	26.82 Humulene<alpha->	0	1.1	0.7	1.9	0.5	0.9
35.57	27.07 Aromadendrene<allo->	0	0.9	0	1.6	0	0.5
36.28	28.15 Germacrene D	4.6	2.1	2.8	2.0	0	2.3
37.37	29.35 Cadinene<gamma->	1.5	1.3	0.6	2.7	1.8	1.6
37.61	29.72 Cadinene<delta->	1.3	2.3	0.5	2.7	0	1.4

Las-/SA-/DAY 1

		% Composition/Collection date					
R.T.	Library/ID	May 19-20	May 25-26	June 2-3	June 8-9	June 16-17	Mean
14.55	Heptanal	1.4	0	2.5	5.7	0	1.9
15.67	α-Thujene	0.8	0	1.3	0	0	0.4
16.00	α-Pinene	1.5	1.0	0	0	1.5	0.8
17.01	Benzaldehyde	0.5	0.4	0.6	0.2	0.9	0.5
17.53	Sabinene	17.8	6.7	11.4	0	5.9	8.3
17.72	β-Pinene	1.6	1.1	1.5	0	1.9	1.2
17.90	6-methyl-5-Hepten-2-one	0	1.7	0.0	0	0	0.3
18.08	Myrcene	4.3	2.5	3.6	0	0	2.1
18.52	Octanal	0	2.8	2.4	5.6	3.7	2.9
18.63	(z)-3-Hexenyl acetate	3.2	1.1	1.0	0	0	1.1
18.72	α-Phellandrene	1.1	0.9	1.2	0	0	0.6
18.96	δ-3-Carene	7.8	2.9	3.9	1.5	2.9	3.8
19.18	α-terpinene	0.4	0.4	0.6	0	1.2	0.5
19.48	p-Cymene	1.1	1.0	1.4	3.7	2.5	1.9
19.65	Limonene	8.0	5.0	3.4	4.5	6.1	5.4
19.86	Z-β-Ocimene	0.6	1.1	1.0	0	0	0.5
20.27	E-β-Ocimene	15.2	38.0	29.4	28.5	17.0	25.6
20.75	Y-Terpinene	0.8	0.6	1.0	0	2.1	0.9
21.08	E-Sabinene hydrate	1.2	0.6	1.2	0	2.3	1.0
21.85	Terpinolene	2.0	0.9	1.4	0	1.4	1.1
22.14	Linalool	5.3	1.9	3.5	0	0	2.1
22.28	Nonanal	4.3	11.3	6.8	27.5	17.2	13.4
23.18	allo-Ocimene	0.4	1.0	0.7	0	0.5	0.5
24.98	Terpinen-4-ol	0	0	0	0	1.7	0.3
25.54	Methyl Salicylate	1.6	0.4	0.5	1.6	1.1	1.1
25.69	Decanal	3.7	6.0	6.8	16.7	8.6	8.3
26.88	Neral	1.4	0.4	0	0	0	0.4
27.37	Car-3-en-2-one	0.4	0	0	0	1.1	0.3
27.77	Geranial	2.7	0.8	0	0	0	0.7
28.71	Indole	0.8	0.4	0.8	2.1	0.0	0.8
28.92	Undecanal	1.0	0.9	1.0	2.6	2.4	1.6
32.59	β-Elemene	1.7	0.6	1.5	0	0	0.8
32.88	Dodecanal	0.3	0	0.5	0	0.7	0.3
33.92	Caryophyllene	3.2	2.6	4.7	0	11.9	4.5
34.25	(-)-γ-elemene	0	0.1	0	0	0	0.0
34.68	Geranyl acetone	0.9	1.5	0.8		2.1	1.3
34.84	E-β-farnesene	0.6	0.7	0.7	0	0	0.4
35.23	α-Humulene	0.6	0	0.4	0	0	0.2
36.22	Germacrene D	0	1.3	0	0	0	0.3
36.75	alpha-Muurolene	0.9	0	0	0	0	0.2
37.31	Y-Cadinene	0.4	0.8	1.3	0	2.3	0.9
37.55	δ-Cadinene	0.5	0.7	1.1	0	1.1	0.7

Las-/SA+/DAY 1

% Composition/Collection date

R.T.	Library/ID	May 19-20	May 25-26	June 2-3	June 8-9	June 16-17	Mean
14.55	Heptanal	0.3	0	0.8	0	0	0.2
15.68	α-Thujene	0.0	0	0.0	0.4	0	0.1
16.00	α-Pinene	0.2	0	0.3	0.8	0.1	0.3
17.01	6.43 Benzaldehyde	0.1	0.2	0.2	0.0	0.5	0.2
17.53	Sabinene	1.8	1.0	2.4	10.7	0	3.2
17.72	β-Pinene	0.3	0	0.4	0.7	0	0.3
17.90	6-methyl-5-Hepten-2-one	0.0	1.6	0.0	1.9	0	0.7
18.08	Myrcene	0.7	0	0.0	0	0	0.1
18.52	Octanal	0.3	1.5	0.9	0	0	0.5
18.63	(z)-3-Hexenyl acetate	0.7	3.4	0.3	0.9	0.2	1.1
18.72	α-Phellandrene	0.2	0	0.4	0.6	0.2	0.3
18.96	δ-3-Carene	0.8	0.7	1.3	5.8	0.2	1.7
19.18	α-terpinene	0.1	0	0.1	0.2	0.1	0.1
19.48	p-Cymene	0.2	0.5	0.4	0.5	0.2	0.3
19.65	Limonene	0.7	0.8	2.7	13.2	0.4	3.6
18.72	β-Phellandrene	0.0	0	0.0	0	0	0
19.86	Z-β-Ocimene	0.3	0.5	0.2	0.3	0.2	0.3
20.27	E-β-Ocimene	8.7	13.4	5.3	8.4	2.9	7.7
20.75	ϒ-Terpinene	0.1	0.3	0.2	0.2	0.1	0.2
21.08	E-Sabinene hydrate	0.2	0.2	0.2	0.3	0.3	0.2
21.85	Terpinolene	0.2	0	0.4	1.2	0.1	0.4
22.14	Linalool	0.7	0.9	1.0	2.7	0.7	1.2
22.28	Nonanal	1.7	7.9	4.0	0.6	1.6	3.2
23.18	allo-Ocimene	0.2	0.4	0.2	0.2	0.1	0.2
25.43	α-Terpineol	0.0	0.0	0.0	0.3	0	0.1
25.54	Methyl Salicylate	80.3	59.0	72.1	43.6	90.4	69.1
25.69	Decanal	0.0	3.6	4.0	0	0	1.5
26.45	Nerol	0	0.0	0.0	0.3	0	0.1
26.88	Neral	0.1	0.7	0.4	0.9	0.2	0.4
27.21	Geraniol	0	0.0	0.0	0.2	0	0.04
27.37	Car-3-en-2-one	0.0	0.0	0.0	0.1	0.1	0.04
27.77	18.62 Geranial	0.4	1.2	0.9	1.2	0.3	0.8
28.71	Indole	0.04	0	0.1	0.05	0	0.03
28.92	Undecanal	0.2	0.6	0.4	0.1	0.2	0.3
32.00	α-Copaene	0	0	0	0.1	0	0.02
32.59	β-Elemene	0.1	0	0.0	1.0	0	0.2
32.88	Dodecanal		0	0.3	0	0.1	0.1
33.92	Caryophyllene	0.4	0.3	0.1	1.6	1.0	0.7
34.68	Geranyl acetone	0.1	0.5	0.1	0.1	0	0.2
35.01	β-Sesquiphellandrene	0	0	0	0.1	0	0.01
34.84	E-β-farnesene	0	0	0.0	0.2	0	0.04
35.23	α-Humulene	0	0	0.0	0.2	0	0.05
36.74	α-Muurolene	0	0	0	0.2	0	0.04
37.31	ϒ-Cadinene	0	0.4	0.0	0	0	0.1
37.55	δ-Cadinene	0	0.2	0.0	0.1	0.05	0.1

Las+/SA-/DAY 1

% Composition/Collection date

R.T.	Library/ID	May 19-20	May 25-26	June 2-3	June 8-9	June 16-17	Mean
14.55	Heptanal	0	0	4.8	1.3	0	1.2
15.68	α-Thujene	0	0	5.6	0	0	1.1
16.00	α-Pinene	0	0.7	1.8	0	1.4	0.8
17.01	6.43 Benzaldehyde	0.8	0.4	0.6	0.05	0.3	0.4
17.53	Sabinene	1.9	4.8	2.6	0	10.0	3.9
17.72	β-Pinene	0	0.9	1.7	0	1.5	0.8
18.08	Myrcene	0	0	0	0	3.1	0.6
18.52	Octanal	2.9	0	3.3	0	1.4	1.5
18.63	(z)-3-Hexenyl acetate	0	0.7	0	0	2.8	0.7
18.72	α-Phellandrene	0	0.7	7.2	0	1.1	1.8
18.96	δ-3-Carene	0.4	1.0	0	0	4.3	1.1
19.18	α-terpinene	0	0.6	0.8	0	0.5	0.4
19.48	p-Cymene	0.7	1.1	10.4	0	1.0	2.6
19.65	Limonene	1.0	2.1	3.7	1.1	3.7	2.3
18.72	β-Phellandrene	1.1	0	6.7	0	0	1.6
19.86	Z-β-Ocimene	26.5	0.5	0.8	0	1.2	5.8
20.27	E-β-Ocimene	0	12.7	17.8	0.4	28.3	11.9
20.75	Ɣ-Terpinene	0.5	0.8	1.0	0	1.1	0.7
21.08	E-Sabinene hydrate	0	0.7	0.8	0	1.2	0.5
21.85	Terpinolene	0	0.6	0	0	1.3	0.4
22.14	Linalool	1.2	1.9	0	8.0	3.1	2.8
22.28	Nonanal	13.6	7.1	18.0	11.9	10.3	12.2
23.18	allo-Ocimene	0	0.5	0.6	0	1.1	0.4
24.98	Terpinen-4-ol	0	1.2	0	0	0.7	0.4
25.54	Methyl Salicylate	2.2	46.7	0.3	0.3	10.0	11.9
25.69	Decanal	7.8	3.5	6.4	13.0	3.8	6.9
26.46	Nerol	0	0	0	1.8	0	0.4
26.88	Neral	0	0.2	0	4.9	0	1.0
27.37	Car-3-en-2-one	0	0.1	0	0	0.4	0.1
27.77	Geranial	0	0.3	0	11.3	1.6	2.7
28.71	Indole	1.7	0	0.7	2.7	0	1.0
28.92	Undecanal	1.5	0.7	0.8	2.4	0	1.1
32.59	β-Elemene	0	0	0.0	10.7	0.7	2.3
32.88	Dodecanal	0.3	0.4	0.6	0	0.2	0.3
33.47	Z-α-Bergamotene	0	2.1	0	0	0	0.4
33.92	Caryophyllene	31.8	3.6	1.7	11.4	2.2	10.1
34.68	Geranyl acetone	1.2	0.7	0.5	0	0	0.5
34.84	E-β-farnesene	0	0.1	0	6.5	0	1.3
35.01	Sesquisabinene	0	0.4	0	0	0	0.1
35.23	α-Humulene	1.6	0.2	0	3.4	0	1.0
36.74	α-Muurolene	0	0	0	6.1	0.9	1.4
36.91	Bisabolene<beta->	0	0.4	0	0	0	0.1
37.31	Ɣ-Cadinene	0.7	0	0.6	1.1	0.3	0.5
37.55	δ-Cadinene	0.6	0	0.5	1.6	0.4	0.6

Las+/SA+/DAY 1

% Composition/Collection date

R.T.	Library/ID	May 19-20	May 25-26	June 2-3	June 8-9	June 16-17	Mean
13.38	Butyl aldoxime, 2-methyl-	0.01	0	0	0	0	t
14.55	Heptanal	0	0	0	0	0	0
15.68	α-Thujene	0	0	0	0.3	0	0.1
16.00	α-Pinene	0.1	0.7	0	0.4	0.4	0.3
17.01	6.43 Benzaldehyde	0.1	0.2	0.2	0.0	0.1	0.1
17.53	Sabinene	1.7	3.8	0.9	4.4	4.4	3.0
17.72	β-Pinene	0	0.7	1.0	0.5	0.4	0.5
17.89	6-Methyl-5-Hepten-2-one	0	2.0	0	0	0	0.4
18.08	Myrcene	0.8	1.5	0	1.4	1.2	1.0
18.52	Octanal	0.2	1.9	0.6	0	0	0.6
18.63	(z)-3-Hexenyl acetate	0.2	0.7	0	1.7	0	0.5
18.72	α-Phellandrene	0	0.7	0.2	0.4	0.3	0.3
18.96	δ-3-Carene	0.2	1.3	0.1	2.3	1.5	1.1
19.18	α-terpinene	0.1	0	0.4	0.1	0.1	0.2
19.48	p-Cymene	0.2	1.1	0.5	0.5	0.2	0.5
19.65	Limonene	0.5	2.3	0	2.3	1.3	1.3
18.72	β-Phellandrene	0	0	0.2	0	0	0
19.86	Z-β-Ocimene	0.5	0	0.6	0.6	0.3	0.4
20.27	E-β-Ocimene	14.7	10.7	17.0	18.5	7.6	13.7
20.75	ϒ-Terpinene	0.2	0.4	0.2	0.2	0.3	0.2
21.08	E-Sabinene hydrate	0.2	0	0.2	0.3	0.6	0.3
21.85	Terpinolene	0.1	0	0	0.5	0.3	0.2
22.14	Linalool	0.4	0.7	0	1.0	3.0	1.0
22.28	Nonanal	1.0	8.7	2.5	1.1	2.0	3.0
23.18	allo-Ocimene	0.3	0	0.4	0.4	0.2	0.3
25.00	14.66 Terpinen-4-ol	0.2	0	0	0	0	0
25.46	alpha.-Terpineol	0	0	0	0	0.4	0.1
25.54	Methyl Salicylate	75.5	59.2	70.6	57.6	72.1	67.0
25.69	Decanal	0	3.1	2.2	1.1	0	1.3
26.46	1Nerol	0	0	0	0	0.2	0
26.88	Neral	0	0	0	0.6	0.4	0.2
27.22	Linalyl acetate	0	0	0	0	0.2	0
27.37	Car-3-en-2-one	0	0	0	0	0.1	0
27.77	Geranial	0	0	0	1.1	0.7	0.4
28.71	Indole	0.1	0	0.1	0.1	0	0.1
28.92	Undecanal	0.2	0	0.3	0.1	0.2	0.1
32.01	Copaene	0	0	0	0.02	0	0
32.59	β-Elemene	0	0	0	0.3	0	0.1
32.88	Dodecanal	0.2	0.4	0.2	0	0.1	0.2
33.47	Z-α-Bergamotene	0.2	0	0	0	0	0
33.92	Caryophyllene	1.7	0	1.4	1.3	0.9	1.0
34.68	Geranyl acetone	0.1	0	0.2	0.2	0.2	0.1
34.84	E-β-farnesene	0	0	0	0.2	0	0
35.23	α-Humulene	0.2	0	0	0.2	0	0.1
35.50	alpha-Z-Bergamotene	0.3	0	0	0	0	0.1
36.74	α-Muurolene	0.0	0	0	0.4	0	0.1
36.92	Bisabolene<beta->	0.03	0	0	0	0	0
37.31	ϒ-Cadinene	0.1	0	0	0	0.1	0
37.55	δ-Cadinene	0.1	0	0	0.1	0.1	0.1

Las-/SA-/DAY 2

% Composition/Collection Date

RT	Compound	May 19-20	May 25-26	June 2-3	June 8-9	June 16-17	Mean
15.67	α-Thujene	2.9	0	3.5	0	0	1.3
15.99	α-Pinene	2.3	2.0	2.5	4.3	2.0	2.6
17.00	Benzaldehyde	0.2	1.1	1.7	1.4	1.2	1.1
17.53	Sabinene	4.7	3.8	6.4	6.6	8.1	5.9
17.71	β-Pinene	2.8	2.3	2.6	0	0	1.5
18.08	Myrcene	0	0	4.3	0	0	0.9
18.52	Octanal	0	5.0	3.1	16.0	3.4	5.5
18.72	α-Phellandrene	3.6	0	4.4	0	3.3	2.3
18.96	δ-3-Carene	2.6	1.7	3.3	2.8	2.6	2.6
19.18	α-Terpinene	1.7	1.1	1.3	0	2.2	1.3
19.47	p-Cymene	10.9	1.8	6.4	4.2	6.9	6.0
19.65	Limonene	1.0	1.3	3.0	6.5	8.5	4.1
19.69	β-Phellandrene	2.8	0	3.3	0	0	1.2
19.86	Z-β-Ocimene	0	1.0	1.0	0	0	0.4
20.26	E-β-Ocimene	11.6	26.0	13.0	7.9	7.5	13.2
20.75	Y-Terpinene	2.8	1.8	2.1	2.2	3.9	2.6
21.08	E-Sabinene hydrate	1.9	1.2	1.3	1.0	1.9	1.5
21.85	Terpinolene	1.7	1.4	1.5	0	2.0	1.3
22.14	Linalool	3.7	0	1.3	0	2.7	1.5
22.28	Nonanal	19.3	20.6	12.7	35.5	19.5	21.5
23.19	allo-Ocimene	0	0.8	0	0	0	0.2
24.98	Terpinen-4-ol	0	0	1.2	0	0	0.2
25.68	Methyl Salicylate	4.7	2.9	1.2	0.7	1.2	2.1
25.68	Decanal	16.4	13.9	10.4	10.2	9.4	12.1
28.92	Undecanal	0	1.4	1.4	0.7	0.5	0.8
32.88	Dodecanal	0	0.9	0.5	0	0	0.3
33.92	Caryophyllene	2.5	4.6	4.7	0	7.9	3.9
34.68	Geranyl acetone	0.003	1.1	0	0	1.0	0.4
34.84	E-β-farnesene	0	0.6	0	0	0	0.1
35.22	α-Humulene	0	0	0	0	0.3	0.1
37.32	Y-Cadinene	0	0.9	1.0	0	1.7	0.7
37.55	δ-Cadinene	0	0.9	0.9	0	1.9	0.7

Las-/SA+/DAY 2

% Composition/Collection Date

RT	Compound	May 19-20	May 25-26	June 2-3	June 8-9	June 16-17	Mean
15.99	α-Pinene	1.9	0.9		1.1	1.3	1.3
17.00	Benzaldehyde	0.1	0.7	1.9	0.3	0.5	0.7
17.53	Sabinene	5.7	3.1	3.2	7.0	7.7	5.3
17.71	β-Pinene	2.1	0	2.5	1.1	1.3	1.4
18.08	Myrcene	0	0	0	2.4	2.2	0.9
18.52	Octanal	5.1	2.2	2.2	2.9	1.5	2.8
18.64	(z)-3-Hexenyl acetate	1.2	1.1	0	0	0	0.5
18.72	α-Phellandrene	1.5	0	3.6	0	1.8	1.4
18.96	δ-3-Carene	3.7	1.5	1.6	5.0	3.9	3.1
19.18	α-Terpinene	0.8	0.3	0.5	0.4	0.8	0.6
19.47	p-Cymene	5.7	0.8	5.0	1.9	3.9	3.5
19.65	Limonene	3.7	1.8	3.4	8.6	4.8	4.5
19.69	β-Phellandrene	0	0	2.2	0	1.8	0.8
19.86	Z-β-Ocimene	0	0.5	0	0.8	0.5	0.4
20.26	E-β-Ocimene	6.0	11.5	5.2	8.2	8.3	7.8
20.75	Y-Terpinene	2.2	0.8	1.3	0.7	1.3	1.3
21.08	E-Sabinene hydrate	1.7	0	1.4	0	0.6	0.8
21.85	Terpinolene	1.0	0	0	0.8	0.9	0.6
22.14	Linalool	0	0	0	0	0.8	0.2
22.28	Nonanal	16.6	8.3	10.0	5.6	7.5	9.6
23.19	allo-Ocimene		0.3	0	0.3	0.4	0.2
24.98	Terpinen-4-ol	1.3	0	0	0	1.0	0.4
25.68	Methyl Salicylate	21.0	56.9	44.9	44.8	35.5	40.6
25.68	Decanal	11.6	5.9	7.0	3.8	6.1	6.9
27.37	Car-3-en-2-one	0.8	0.5	0	0.4	0.3	0.4
28.92	Undecanal	1.8	0.8	1.7	0	1.1	1.1
32.88	Dodecanal		0.5	0.7	0.6	1.0	0.7
33.92	Caryophyllene	2.1	1.2	0	2.3	2.1	1.5
34.68	Geranyl acetone	0.1	0.7	1.9	0.4	0	0.6
37.32	Y-Cadinene	0	0.4	0	0	0	0.1
37.55	δ-Cadinene	2.2	0.4	0	0.5	1.0	0.8

Las+/SA-/DAY 2

RT	Compound	% Composition/Collection date					
		May 19-20	May 25-26	June 2-3	June 8-9	June 16-17	Mean
15.67	α-Thujene	1.8	0	0	0	2.0	0.8
16.03	α-Pinene	0	0	0	4.8	1.2	1.2
16.99	Benzaldehyde	0.3	0.4	2.1	1.7	8.1	2.5
17.00	Sabinene	1.1	3.7	2.3	8.3	0	3.1
17.53	β-Pinene	0	1.0	3.4	0	0	0.9
17.71	Octanal	7.1	1.6	3.4	0	3.4	3.1
18.64	(z)-3-Hexenyl acetate	0	0	1.2	0	0	0.2
18.72	α-Phellandrene	4.7	0	9.8	0	3.3	3.6
18.96	δ-3-Carene	0	0.9	1.0	2.8	2.6	1.5
19.18	α-Terpinene	0	0.6	1.6	0	2.2	0.9
19.47	p-Cymene	11.3	1.9	14.8	3.7	6.9	7.7
19.65	Limonene	1.4	2.2	4.8	5.1	8.5	4.4
19.69	β-Phellandrene	5.0	0	8.5	0	0	2.7
19.86	Z-β-Ocimene	0	0.5	0	0	0	0.1
20.26	E-β-Ocimene	8.7	7.6	9.3	6.8	7.5	8.0
20.75	Ɣ-Terpinene	0	1.1	2.9	3.2	3.9	2.2
21.08	E-Sabinene hydrate	0	0.8	1.8	2.3	1.9	1.4
21.85	Terpinolene	0	0	0	0	2.0	0.4
22.14	Linalool	0	0	0	0	2.7	0.5
22.28	Nonanal	22.9	10.0	18.8	40.8	19.5	22.4
24.98	Terpinen-4-ol	0	0	1.9	0	0	0.4
25.39	α-Terpineol	0	1.4	0	0	0	0.3
25.68	Methyl Salicylate	2.6	50.7	3.5	1.6	1.2	11.9
25.68	Decanal	12.5	4.9	2.6	14.2	9.4	8.7
27.37	Car-3-en-2-one	0	0	0	0	0.5	0.1
28.92	Undecanal	2.6	0.8	0	1.9	0.5	1.2
32.88	Dodecanal	1.9	0.4	0	0	0	0.5
33.47	Z-α-Bergamotene	0	3.5	0	0	0	0.7
33.92	Caryophyllene	11.6	2.7	3.4	1.9	7.9	5.5
34.68	Geranyl acetone	0	1.3	0	0	1.0	0.5
35.01	Sesquisabinene	0	0.7	0	0	0	0.1
35.23	α-Humulene	0	0	0	0	0.3	0.1
35.91	β-Bisabolene	0	0.8	0	0	0	0.2
37.32	Ɣ-Cadinene	0	0.3	1.5	0	1.7	0.7
37.55	δ-cadinene	4.7	0	1.4	1.0	1.9	1.8

Las+/SA+/DAY 2

RT	Compound	% Composition/Collection date May 19-20	May 25-26	June 2-3	June 8-9	June 16-17	Mean
16.03	α-Pinene	0	2.3	0	1.4	1.3	1.0
16.99	Benzaldehyde	0.1	1.1	0.6	0.3	0.5	0.5
17.00	Sabinene	2.9	7.5	4.7	5.5	7.7	5.6
17.53	β-Pinene	0	2.5	1.1	1.6	1.3	1.3
18.12	Myrcene	0	0	0	0	2.2	0.4
17.71	Octanal	0	5.0	2.2	3.7	1.5	2.5
18.64	(z)-3-Hexenyl acetate	0	0.9	0	1.1	0	0.4
18.72	α-Phellandrene	1.0	0	0	0	1.8	0.6
18.96	δ-3-Carene	0.5	2.5	1.5	2.5	3.9	2.2
19.18	α-Terpinene	0	0	0.3	0.7	0.8	0.4
19.47	p-Cymene	0	3.3	1.4	1.7	3.9	2.1
19.65	Limonene	1.2	5.4	5.0	5.4	4.8	4.4
19.69	β-Phellandrene	0.6	0	0	0	1.8	0.5
19.86	Z-β-Ocimene	0	0.6	0.7	0	0.5	0.4
20.26	E-β-Ocimene	8.4	9.7	13.3	19.2	8.3	11.8
20.75	Ɣ-Terpinene	0.9	2.0	0	1.1	1.3	1.1
21.08	E-Sabinene hydrate	0	1.5	0.7	1.0	0.6	0.8
21.85	Terpinolene	0.2	0	0	1.1	0.9	0.4
22.14	Linalool	1.1	0	0.9	0	0.8	0.6
22.28	Nonanal	6.9	22.2	6.3	5.4	7.5	9.7
23.2	allo-Ocimene	0.2	0.6	0.4	0.7	0.4	0.5
24.98	Terpinen-4-ol	0.5	0	0	0	1.0	0.3
25.68	Methyl Salicylate	62.9	14.6	52.5	36.9	35.5	40.5
25.68	Decanal	5.9	14.2	3.9	3.8	6.1	6.8
27.37	Car-3-en-2-one		0.6	0.3	0.4	0.3	0.4
28.75	Indole	0	0	0	0.3	0	0.1
28.92	Undecanal	0	1.7	0.6	0.5	1.1	0.8
32.88	Dodecanal		1.1	0.3	0.8	1.0	0.8
33.47	Z-α-Bergamotene	1.3	0	0	0	0	0.3
33.92	Caryophyllene	5.3	0	2.5	3.4	2.1	2.7
34.68	Geranyl acetone	0	0	0.8	1.0	0	0.4
37.32	Ɣ-Cadinene	0	0	0	0.5	0	0.1
37.55	δ-Cadinene	0	0	0	0.4	1.0	0.3
