# Supplementary material for: Exogenous application of the plant signalers methyl jasmonate and salicylic acid induces changes in volatile emissions from citrus foliage and influences the aggregation behavior of Asian citrus psyllid (Diaphorina citri), vector of Huanglongbing
Source: PLoS One. 2018 Mar 29;13(3):e0193724. doi: 10.1371/journal.pone.0193724 (PMC5875780; doi:10.1371/journal.pone.0193724)
Supplement: S1 Table — Data were square root transformed prior to analysis. (PDF) [file pone.0193724.s002.pdf]

S2 Table. Permutational MANOVA analysis of the effects of Las infection status, methyl jasmonate application, and collection day (Day 1 v. Day 2) on the proportions of volatile compounds emitted from Valencia sweet orange. Data were square root transformed prior to analysis.

Source	df	SS	MS	Psuedo-F	P(perm)	Unique permuations
Las	1	2641	2641	5.9483	0.0003	9936
MJ	1	6984	6982	15.7290	0.0001	9923
Collection Day	1	3722	3722	8.3814	0.0001	9933
Las x MJ	1	834	834	1.8794	0.0720	9931
Las x Day	1	191	191	0.4305	0.8900	9945
MJ x Day	1	965	965	2.1728	0.0379	9932
Las x MJ x Day	1	512	512	1.1534	0.3146	9935
Residual	31	13764	444			
Total	38	29209				
