# Supplementary material for: Exogenous application of the plant signalers methyl jasmonate and salicylic acid induces changes in volatile emissions from citrus foliage and influences the aggregation behavior of Asian citrus psyllid (Diaphorina citri), vector of Huanglongbing
Source: PLoS One. 2018 Mar 29;13(3):e0193724. doi: 10.1371/journal.pone.0193724 (PMC5875780; doi:10.1371/journal.pone.0193724)
Supplement: S2 Table — Data were square root transformed prior to analysis. (PDF) [file pone.0193724.s003.pdf]

**S3 Table. Permutational MANOVA analysis of the effects of Las infection status, methyl jasmonate application, and collection day (Day 1 v. Day 2) on the proportions of chemical classes emitted from Valencia sweet orange. Data were square root transformed prior to analysis.**

| Source | df | SS | MS | Psuedo-F | P(perm) | Unique permuations |
|---|---|---|---|---|---|---|
| **Las** | 1 | 1926.4 | 1930.0 | 13.13 | 0.0001 | 9963 |
| **MJ** | 1 | 5208.6 | 5210.0 | 35.501 | 0.0001 | 9946 |
| **Collection Day** | 1 | 1368.8 | 1368.8 | 9.3295 | 0.0007 | 9962 |
| **Las x MJ** | 1 | 497.62 | 497.6 | 3.3917 | 0.0452 | 9956 |
| **Las x Day** | 1 | 94.63 | 94.6 | 0.64498 | 0.5539 | 9957 |
| **MJ x Day** | 1 | 139.46 | 139.4 | 0.95057 | 0.4148 | 9957 |
| **Las x MJ x Day** | 1 | 70.914 | 70.9 | 0.48334 | 0.6379 | 9962 |
| **Residual** | 31 | 4694.9 | 146.72 | | | |
| **Total** | 38 | 14001 | | | | |
