# Supplementary material for: Exogenous application of the plant signalers methyl jasmonate and salicylic acid induces changes in volatile emissions from citrus foliage and influences the aggregation behavior of Asian citrus psyllid (Diaphorina citri), vector of Huanglongbing
Source: PLoS One. 2018 Mar 29;13(3):e0193724. doi: 10.1371/journal.pone.0193724 (PMC5875780; doi:10.1371/journal.pone.0193724)
Supplement: S3 Table — AA: alkyl aldehydes, MT: monoterpenes, ST: sesquiterpenes, N: Nitrogen-compounds, E-J: Z- jasmone, MeJA: Methyl salicylate. (PDF) [file pone.0193724.s004.pdf]

S4 Table. Total amount (ng) of each chemical class and selected compounds emitted in the MJ Experiment (n = 5 replicates per treatment). AA: alkyl aldehydes, MT: monoterpenes, ST: sesquiterpenes, N: Nitrogen-compounds, E-J: Z- jasmone, MeJA: Methyl salicylate.

Total amount (ng) emitted per treatment

DAY 1

	Total	AA	MT	ST	N	Z-J	MeSA
**Las-/MJ-**	1461	594	761	70	3	0	9
**Las+/MJ-**	3142	716	2071	227	10	0	141
**Las-/MJ+**	6212	777	4839	260	275	23	15
**Las+/MJ+**	7641	660	6655	725	281	41	37

DAY 2

	Total	AA	MT	ST	N	Z-J	MeSA
**Las-/MJ-**	621	418	169	33	0	0	7
**Las+/MJ-**	1158	422	494	74	0	0	105
**Las-/MJ+**	2737	566	1869	236	37	1	29
**Las+/MJ+**	2333	418	1283	579	46	0	37
