# Supplementary material for: Exogenous application of the plant signalers methyl jasmonate and salicylic acid induces changes in volatile emissions from citrus foliage and influences the aggregation behavior of Asian citrus psyllid (Diaphorina citri), vector of Huanglongbing
Source: PLoS One. 2018 Mar 29;13(3):e0193724. doi: 10.1371/journal.pone.0193724 (PMC5875780; doi:10.1371/journal.pone.0193724)
Supplement: S4 Table — Data were square root transformed prior to analysis. (PDF) [file pone.0193724.s005.pdf]

S5 Table. Permutational MANOVA analysis of the effects of Las infection status, salicylic acid application, and collection day (Day 1 v. Day 2) on the proportions of volatile compounds emitted from Valencia sweet orange. Data were square root transformed prior to analysis.

Source	df	SS	MS	Psuedo-F	P(perm)	Unique permuations
**Las**	1	294	294	0.5897	0.7700	9948
**SA**	1	4137	4137	8.2974	0.0001	9944
**Collection Day**	1	3461	3461	6.9403	0.0001	9951
**Las x SA**	1	369	369	0.7415	0.6527	9949
**Las x Day**	1	73	73	0.1474	0.9801	9932
**SA x Day**	1	905	904	1.8146	0.0798	9939
**Las x SA x Day**	1	316	316	0.6342	0.7386	9941
**Residual**	31	15958	498			
**Total**	38	25515				
