# Supplementary material for: Exogenous application of the plant signalers methyl jasmonate and salicylic acid induces changes in volatile emissions from citrus foliage and influences the aggregation behavior of Asian citrus psyllid (Diaphorina citri), vector of Huanglongbing
Source: PLoS One. 2018 Mar 29;13(3):e0193724. doi: 10.1371/journal.pone.0193724 (PMC5875780; doi:10.1371/journal.pone.0193724)
Supplement: S5 Table — Data were square root transformed prior to analysis. (PDF) [file pone.0193724.s006.pdf]

S6 Table. Permutational MANOVA analysis of the effects of Las infection status, salicylic acid application, and collection day (Day 1 v. Day 2) on the proportions of chemical classes emitted from Valencia sweet orange. Data were square root transformed prior to analysis.

Source	df	SS	MS	Psuedo-F	P(perm)	Unique permuations
**Las**	1	179.5	179.58	0.90395	0.4676	9957
**MJ**	1	10055.0	10055	50.615	0.0001	9954
**Collection Day**	1	1043.6	1043.6	5.2532	0.0033	9967
**Las x MJ**	1	338.2	338.19	1.7024	0.1872	9960
**Las x Day**	1	56.8	56.763	0.28573	0.8148	9955
**MJ x Day**	1	935.0	934.97	4.7064	0.0049	9959
**Las x MJ x Day**	1	-86.0	-86.017	Negative		
**Residual**	31	6357	198.66			
**Total**	38	18879				
