# Supplementary material for: Exogenous application of the plant signalers methyl jasmonate and salicylic acid induces changes in volatile emissions from citrus foliage and influences the aggregation behavior of Asian citrus psyllid (Diaphorina citri), vector of Huanglongbing
Source: PLoS One. 2018 Mar 29;13(3):e0193724. doi: 10.1371/journal.pone.0193724 (PMC5875780; doi:10.1371/journal.pone.0193724)
Supplement: S6 Table — AA: alkyl aldehydes, MT: monoterpenes, ST: sesquiterpenes, N: Nitrogen-compounds, MeJA: Methyl salicylate. (PDF) [file pone.0193724.s007.pdf]

S7 Table. Total amount (ng) of each chemical class and selected compounds emitted in the SA Experiment (n = 5 replicates per treatment). AA: alkyl aldehydes, MT: monoterpenes, ST: sesquiterpenes, N: Nitrogen-compounds, MeJA: Methyl salicylate.

Total amount (ng) emitted per treatment

DAY 1

	Total	AA	MT	ST	N	MeSA
**Las-/SA-**	2827	604	1890	236	17	25
**Las+/SA-**	2944	592	1300	439	21	624
**Las-/SA+**	16275	653	3994	249	6	11221
**Las+/SA+**	13734	513	3413	233	7	9498

DAY 2

	Total	AA	MT	ST	N	MeSA
**Las-/SA-**	1455	562	769	92	0	34
**Las+/SA-**	1673	503	604	133	0	417
**Las-/SA+**	3122	536	1040	70	0	1443
**Las+/SA+**	3382	633	1198	124	2	1459
